# Classification of Red Wines Produced from Zweigelt and Rondo Grape Varieties Based on the Analysis of Phenolic Compounds by UPLC-PDA-MS/MS

**DOI:** 10.3390/molecules25061342

**Published:** 2020-03-16

**Authors:** Anna Stój, Ireneusz Kapusta, Dorota Domagała

**Affiliations:** 1 Department of Biotechnology, Microbiology and Human Nutrition, Faculty of Food Science and Biotechnology, University of Life Sciences in Lublin, 8 Skromna Street, 20-704 Lublin, Poland; 2 Department of Food Technology and Human Nutrition, College of Natural Science, Rzeszów University, 4 Zelwerowicza Street, 35-601 Rzeszów, Poland; ikapusta@ur.edu.pl; 3 Department of Applied Mathematics and Computer Science, Faculty of Production Engineering, University of Life Sciences in Lublin, 28 Głęboka Street, 20-612 Lublin, Poland; dorota.domagala@up.lublin.pl

**Keywords:** sugars, organic acids, phenolic compounds, authentication, adulteration, false labelling

## Abstract

The authentication of grape variety from which wine is produced is necessary for protecting a consumer from adulteration and false labelling. The aim of this study was to analyze phenolic compounds in red monovarietal wines produced from Zweigelt (*Vitis vinifera*) and Rondo (non-*Vitis vinifera*) varieties while using the UPLC-PDA-MS/MS method and to assess whether these wines can be classified according to grape variety that is based on chemometric analysis. Fifty-five phenolic compounds belonging to five classes—anthocyanins, flavonols, flavan-3-ols, phenolic acids, and stilbenes—were identified and quantified in Zweigelt and Rondo wines. The wines of the Zweigelt variety were characterized by lower concentrations of phenolic compounds than those of the Rondo variety. Furthermore, wines of the Zweigelt variety contained the highest concentrations of flavan-3-ols, and wines of the Rondo variety—the highest concentrations of anthocyanins. Hierarchical cluster analysis (HCA) revealed that Zweigelt wines and Rondo wines formed two separate groups. The Rondo group was divided into two subgroups, differing in type of malolactic fermentation (spontaneous or induced). Phenolic compounds analysis by means of UPLC-PDA-MS/MS combined with HCA is a useful tool for the classification of red wines that were produced from Zweigelt and Rondo grape varieties, regardless of yeast strain and type of malolactic fermentation.

## 1. Introduction

Wines have high commercial value and they are produced in large volumes, thus they are potentially subjected to adulteration and mislabeling [1]. Typical kinds of wine adulterations are false declaration of variety, geographical origin, vintage, addition of sugar, water, glycerol, and colorants [2,3,4,5,6]. Wine authentication is important to the consumers and industry and it has the purpose of identifying fraud and confirming label declarations. Consumers require that the price of wine is determined by its quality and wine labelling accurately reflects the reliable information. In turn, honest producers expect to ensure fair play on the wine market [1,2,7,8]. Wines contain a large number of metabolites, e.g., sugars, organic acids, amino acids, polyphenols, and aroma compounds [9,10]. Metabolomics studies propose the analysis of metabolites to enable integration of all metabolic changes of wine throughout its processing to assure wine authentication [7].

Wine polyphenols are classified as flavonoids, including anthocyanins, flavan-3-ols, flavonols, flavones, flavanones, isoflavones, and non-flavonoids, comprising phenolic acids and stilbenes [11,12,13]. The phenolic profile of wines depends on the grape variety, the grapes at harvest, geographical origin (environmental conditions, including soil type, climate, solar radiation, altitude), viticultural practices, winemaking techniques, and aging [14,15,16,17]. The grapevine genome affects the concentration and composition of phenols in grapes and the enzymatic reactions that are involved in their biosynthesis are influenced by environmental factors and viticultural practices [18,19]. Polyphenolic profiles can be used as chemical markers of the grape variety [4,8,20,21,22], the geographical origin [4,20,21,23], and vintage [8,20].

Different techniques of liquid chromatography have been used for the analysis of polyphenols in wines. These compounds are usually determined by HPLC with photodiode array detector (HPLC-PDA or DAD, depending on the manufacturer) [24,25,26,27,28,29], UPLC-PDA [30], HPLC coupled with electrospray ionization mass spectrometry (HPLC-ESI-MS) [21], or HPLC with fluorescence detection (HPLC-FLD) [31,32]. Regarding the sample preparation, some authors have used solid-phase extraction (SPE) with different types of cartridges, liquid-liquid extraction (LLE), or injections of the samples directly in HPLC without any preparation [27]. An advanced technologies, such as: LC–MSQTOF [33], LC–MS/MS [20], UPLC-MS/MS [34], UPLC-QTOF [35], and UPLC-PDA-MS/MS [36], several laboratories have recently used for polyphenols analysis. The introduction of UPLC revolutionized separation science. Significant advances in instrumentation and column technology were made to obtain an increase in resolution, speed, and sensitivity in liquid chromatography. A higher separation efficiency of sub-2-µm particle sorbents allows for faster chromatographic separation [30].

Chemometric methods are employed for data processing and they enable the classification of wines according to grape variety. Ma et al. (2014) [25] applied HCA with the use of phenolic components as variables for the successful clustering of red wines that were made of different grape varieties grown in China. A cluster was formed by Cabernet Sauvignon and Merlot wines, another by Cabernet Gernischt and Cinsault wines, and the third cluster by Gamay wines. Some authors applied canonical discriminant analysis (CDA) as a chemometric method for data processing. Jaitz et al. (2010) [20] correctly classified red wines that were produced of Blauer Portugieser, Blauer Wildbacher and Sankt Laurent varieties in 100% and in 65%—of Blauer Zweigelt, Blaufränkisch, and Blauburger varieties. The classification of wines of Blauer Zweigelt, Blaufränkisch, and Blauburger varieties was unacceptable because of low eigenvalues and low canonical correlation. The CDA results of Sun et al. (2015) [4] showed 100% differentiation of red wines (Cabernet Sauvignon, Carmenère, Merlot, Cabernet Franc and Shiraz) according to grape variety. Žurga et al. (2019) [22] successfully used CDA in discriminating Croatian wines that were produced from two native grape varieties (Plavac mali from Teran wines), and their separation from wines of non-native varieties (Cabernet Sauvignon, Merlot). In turn, Ivanova-Petropulos et al. (2015) [24] grouped the red wines of Cabernet Sauvignon, Merlot, Syrah, and Vranac varieties while using PCA. Pisano et al. (2015) [21] showed that multivariate curve resolution–alternating least-squares (MCR-ALS) model allowed for the successful discrimination of red wines of Aspiran, Bonarda, Cabernet Sauvignon, Malbec, Merlot, Sangiovese, Syrah, and Tempranillo varieties, while the discriminant unfolded partial least-squares (D-UPLS) model adequately discriminated Cabernet Sauvignon, Malbec, and Merlot wines from the remaining wines.

There are known cases of false wine labelling with a more expensive grape variety. For example, the producers and trader from the southwest of France were found to be guilty of having supplied an American trader with mislabeled “Pinot Noir” wines [7,37]. When compared to wine countries, such as France, Italy, Spain, or Germany, growing the *Vitis* interspecific hybrids, well-adapted to cold climate, and more resistant to fungal-pathogens, is much more popular in Poland. One of the most-grown red grape hybrid is Rondo (non-*Vitis vinifera*). However, a small area is covered by noble grape varieties, such as Zweigelt (*Vitis vinifera*). The wines that are produced from the noble grape varieties are known for their high quality, but they do not possess a high resistance against fungal infections and winter frost [13,38,39,40,41]. In Poland, there is a possibility to label wine from the Rondo variety as Zweigelt variety to obtain higher profit. The aim of this study was to determine the phenolic compounds in red monovarietal wines from Zweigelt and Rondo varieties while using the UPLC-PDA-MS/MS method and assessing whether these wines can be classified according to grape variety with chemometric analysis. This is the first study regarding the possibility of varietal classification of red wines made in Poland. The study of wines began with determination of oenological parameters, such as: pH, total acidity, sugars, and organic acids, which characterize wines and are directly correlated with its quality and stability.

## 2. Results and Discussion

This paper analyzed twenty wines that were produced from the Zweigelt and Rondo grape varieties. Zweigelt wines, in which alcoholic fermentation was carried out by various yeast strains and malolactic fermentation was spontaneous had Z1–Z5 codes, while Zweigelt wines, in which alcoholic fermentation was carried out by various yeast strains (but the same as in Z1–Z5 wines) and malolactic fermentation was induced had Z1 LAB–Z5 LAB codes. We produced and coded Rondo wines by analogy to Zweigelt wines (R1–R5 and R1 LAB–R5 LAB). We did not identify lactic acid bacteria strains carrying out spontaneous malolactic fermentation in this paper.

Table S1 shows the oenological parameters, such as sugars, organic acids, pH, and total acidity in wines produced from the Zweigelt and Rondo varieties in the Supplementary Materials, and Figures S1–S6 show the chromatograms of sugars and organic acids. These parameters are related to the composition of grapes and changes taking place during vinification. They are used to characterize each wine, because they directly correlate with their quality and stability [36]. Generally, Rondo wines contained similar concentrations of glucose, fructose, tartaric acid, and they had similar values of pH and total acidity to those that were determined by other authors in wines of the same grape variety [34,36,38,42,43]. In addition, they contained higher concentrations of malic acid, acetic acid, citric acid, and lower concentrations of succinic acid. To date, no work has been published containing a value of oenological parameters in Zweigelt wines. In our research, Zweigelt wines contained glucose, while glucose was not detected in Rondo wines. In addition, they were characterized by lower malic acid and higher acetic acid levels.

Wines that were subjected to spontaneous malolactic fermentation (R1–R5 and Z1–Z5) contained more malic acid and less lactic acid, while wines that were subjected to induced malolactic fermentation (R1 LAB–R5 LAB and Z1 LAB–Z5 LAB)—*vice versa*. Thus, induced fermentation was more effective. On the other hand, malic acid has not been completely converted to lactic acid in both types of fermentation. This can be explained by the fact that lactic acid has antimicrobial activity and, at higher concentrations, it might also inhibit the lactic acid bacteria. *O. oeni* activity might be inhibited above a certain lactic acid concentration and, in the case of high-acid musts, a total reduction of malic acid can be impossible [43].

R1–R5 and Z1–Z5 wines contained succinic acid, while R1 LAB–R5 LAB and Z1 LAB–Z5 LAB wines did not have this acid. Based on the literature, one possibility is that all succinic acid was esterified to diethyl succinate in R1 LAB–R5 LAB and Z1 LAB–Z5 LAB wines. Succinic acid is produced during alcoholic fermentation, and it is esterified to diethyl succinate in wines that undergo malolactic fermentation. Diethyl succinate content was much higher in wines that underwent induced malolactic fermentation than in those that underwent spontaneous malolactic fermentation [44].

**Figure 1:**
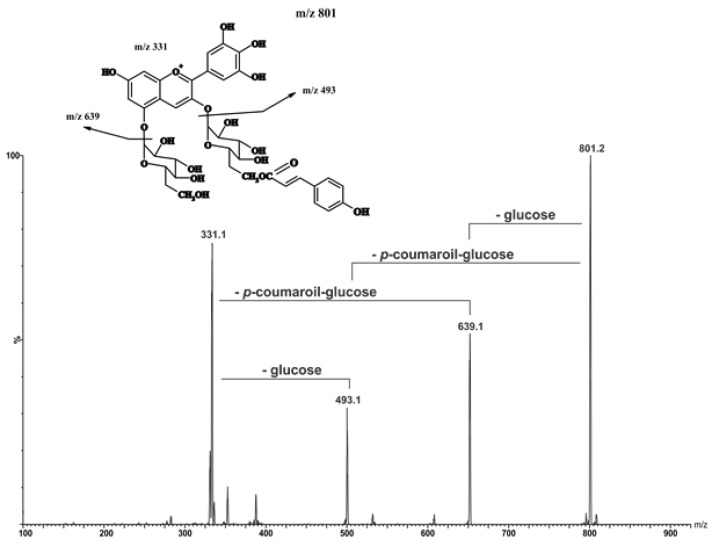
A typical fragmentation pattern mechanism based on the example of malvidine 3-*O*-(6′-*O*-coumaryl)-glucoside-5-*O*-glucoside.

**Figure 2:**
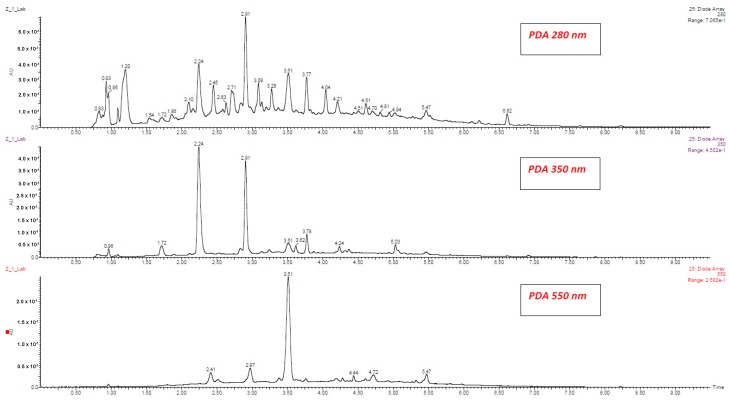
UPLC-PDA chromatograms of phenolic compounds in Zweigelt wine at 280, 350 and 550 nm.

**Figure 3:**
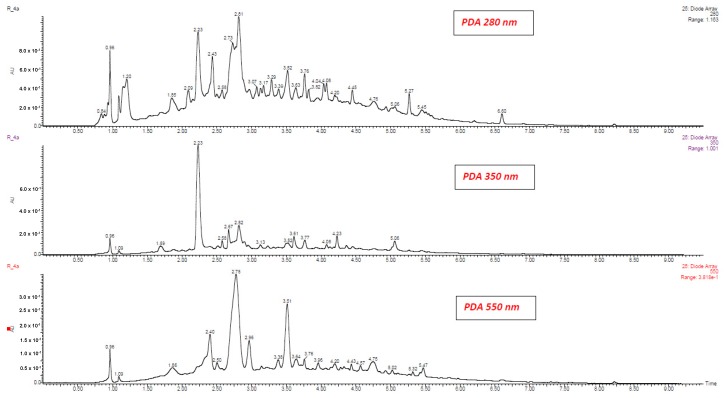
UPLC-PDA chromatograms of phenolic compounds in Rondo wine at 280, 350 and 550 nm.

Table 1, Table S2, and Table S3 show phenolic compounds that were identified and quantified using UPLC-PDA-MS/MS in red wines produced from Zweigelt and Rondo varieties. Figure 1 shows a typical fragmentation pattern mechanism, Figure 2 and Figure 3 show chromatograms of phenolic compounds, and Figures S7–S12 show single ion recording (SIR) of phenolic compounds. The wines of the Zweigelt variety contained lower concentrations of phenolic compounds than those of the Rondo variety. Wines of the Zweigelt variety contained the highest concentrations of flavan-3-ols, and wines of the Rondo variety—the highest concentrations of anthocyanins. The tested wines had average amounts of phenolic acids when compared to other classes of compounds. Flavonols and stilbenes were the minor compounds (Tables S2 and S3).

Anthocyanins constituted the most numerous class among phenolic compounds in wines from both grape varieties. A total of 24 anthocyanins were identified in tested wines, including derivatives of five aglycones: malvidin (5), delphinidin (5), cyanidin (4), peonidin (5), and petunidin (5). Among the compounds, there were mono- and diglucosides, their acylated derivatives, as well as combinations with p-coumaric acid, acetic acid, and caffeic acid. Anthocyanins are water-soluble pigments, which are only present in red grape skins (the entirety of anthocyanins in grapes is made of the derivatives of the five aglycones), being transferred to red wines during vinification (maceration). The presence of anthocyanin diglucosides in red wines, particularly malvidin-3,5-diglucoside, is a quality marker that is used for distinguishing wines produced from *V. vinifera* and non-*V. vinifera* grapes. *V. vinifera* wines contain significant quantities of anthocyanin monoglucosides, on the contrary to non-*V. vinifera* wines, which contain significant quantities of anthocyanin diglucosides [13,36]. In our study, Zweigelt (*V. vinifera*) wines had high concentrations of anthocyanin monoglucosides, such as malvidin 3-*O*-glucoside, delphinidin 3-*O*-glucoside, and petunidin 3-*O*-glucoside, while Rondo (non-*V. vinifera*) wines had high concentrations of malvidin 3-*O*-glucoside-5-*O*-glucoside, peonidin 3-*O*-glucoside-5-*O*-glucoside, and delphinidin 3-*O*-glucoside-5-*O*-glucoside. The anthocyanins diglucosides were also present at a low concentration in Zweigelt wines. The concentration of malvidin 3-*O*-glucoside-5-*O*-glucoside in Zweigelt wines was 0.33 mg/L, which is in accordance with the limit of 15 mg/L that was stated by International Organization of Vine and Wine (OIV) [45]. A previous study of Kapusta et al. (2017) [46] also reported the presence of anthocyanin 3,5-*O*-diglucosides in Zweigelt grapes. Ivanova-Petropulos et al. (2015) [24] proposed the ratio of acetylglucosides and p-coumaroylglucosides (Σ acetylated/Σ coumaroylated) as a verification factor for varietal authenticity of red wines. Values that were calculated by the authors in Macedonian wines ranged among regions: for native Vranec wines 1–1.7, for Syrah 2.6–4.8, for Cabernet Sauvignon 3.2–4.6 and for Merlot wine 2.3–3.8. In our study, Σ acetylated/ Σ coumaroylated ratio for Zweigelt wines was 0.6–1.1 and for Rondo wines, it was 0.7–0.9. Thus, our wines that were produced from grapes grown in Lublin Province cannot be distinguished based on this ratio. In turn, the Σ acetylated/Σ coumaroylated ratio in Rondo wines that were made from grapes growing in Germany was 0.5–1.4 [34].

Twelve compounds that belonged to the class of flavan-3-ols were identified in the wines: two (+) catechin and (−) epicatechin monomers, and ten procyanidins. The flavan-3-ols affect the bitterness, astringency, and structure of wines. Furthermore, they play an important role during ageing, because they stabilize wine color. Flavanols occur in solid parts of grapes (seed, skin, and stem). Like anthocyanins, flavan-3-ols significantly change during maceration and fermentation. The monomeric flavanols easily undergo the oxidation and condensation creating the procyanidin complex, thus decreasing their amount might be observed during crushing of grapes and before the beginning of the fermentation. Flavan-3-ols are extracted during the whole process of maceration and fermentation, in contrast to anthocyanins, which reach their maximum concentration at the beginning of fermentation, because extraction from seeds is slower than from skins. The ethanol concentration has little effect on extraction of flavan-3-ols from skins, while it greatly accelerates the transition of these compounds from seeds to wine [25,36]. In our research, the ratio between (+) catechin and (−) epicatechin in Rondo wines was 2:1, similarly like in wine that is made from the same grape variety studied by Kapusta et al. (2018) [36].

Eight compounds were found in the group of phenolic acids, which were represented by two classes: hydroxycinnamic acids (caftaric, coutaric, caffeic, *p*-coumaric, coumaric, and ferulic) and hydroxybenzoic acids (gallic and protocatechuic). Gallic acid is a major phenolic acid in red wines. The biosynthesis of flavanols influences concentrations of gallic acid in grapes, in particular (+)-gallo(catechins) and (–)-epi(gallo)catechins, which in turn are hydrolyzed to gallic acid, under the action of tannase enzymes. Thus, the grape variety used for a winemaking process and climatic conditions during cultivation have significant influence on the concentration of this phenolic acid [28,36,40]. In our work, wines that are made from the Rondo variety contained higher amounts of gallic acid than Zweigelt wines.

Seven compounds represent the flavonols in the wines, which included derivatives of quercetin (3), myricetin (2), dihydroquercetin (1), and isorhamnetin (1). The flavonols are colorless, but they contribute to the color stabilization of red wines due to co-pigmentation with anthocyanins. They also contribute to wine bitterness [25]. They originate from the grape skins of both white and red grapes. Higher concentrations of flavonols are observed in red wines subjected to maceration [19,25,36]. In our research, the red wines of both varieties mainly contained isorhamnetin 3-*O*-glucoside and myricetin-3-*O*-glucoside.

Stilbenes were the last group of analyzed polyphenolic compounds in the wines. Among this group, the *cis*- and *trans*- isomers of resveratrol and its glycoside derivatives, piceid, were identified. Stilbenes are an important class of phenolic compounds due to their protective effects against cardiovascular diseases [19,25]. Wine is the main source of resveratrol in human diet. The amount of stilbenes in wine varies widely, depending on many factors, including grape variety, plant stress conditions (such as pathogens and ultraviolet light), oenological practices, and enzymatic activity of yeast and lactic acid bacteria [22,28,36]. In our study, the Rondo and Zweigelt wines differed in the content of individual stilbenes, especially *trans*-piceid and *cis*-piceid. Furthermore, the influence of yeast strain and type of malolactic fermentation (spontaneous or induced) on *trans*-piceid content was observed within the wines of the Zweigelt as well as Rondo variety.

The concentrations of the total phenolic compounds in wines that were produced from Zweigelt variety ranged from 128.02 to 273.62 mg/L. While considering concentration of phenolic compounds, wines of the Zweigelt variety contained the highest concentrations of flavan-3-ols, followed by anthocyanins, phenolic acids, stilbenes, and flavonols. The subtotal concentration of flavan-3-ols varied from 68.62 to 132.38 mg/L, for anthocyanins from 22.34 to 120.05 mg/L, for phenolic acids from 24.20 to 33.29 mg/L, for stilbenes from 2.09 to 3.43 mg/L, and for flavonols from 0.32 to 0.70 mg/L. The fraction of flavan-3-ols mainly composed of (−) epicatechin and (+) catechin, anthocyanins—of malvidin 3-*O*-glucoside, phenolic acids—of gallic acid and caftaric acid, stilbenes—of *cis*-resveratrol, and flavonols—of isorhamnetin 3-*O*-glucoside and myricetin-3-*O*-glucoside. Quercetin 3-*O*-glucuronide and dihydroquercetin 3-*O*-ramnoside were not detected in any wine produced from Zweigelt variety. Quercetin 3-*O*-rutinoside was found in two of all ten wines from the Zweigelt variety. A literature review shows that Jaitz et al. (2010) only examined phenolic compounds of Zweigelt wines [20]. The grapes for production of wines were grown in eleven different wine regions in Austria during five vintages from 2003 to 2007. These authors determined the content of several phenolic compounds: gallic acid, caffeic acid, catechin, epicatechin, *cis*-resveratrol, and *trans*-resveratrol. Our Zweigelt wines produced from grapes growing in Poland contained similar amounts of gallic acid, catechin and *cis*-resveratrol to those that were determined by Jaitz et al. (2010) [20]. The differences between the contents of caffeic acid, epicatechin, and *trans*-resveratrol may have been due to different geographical origins and related differences in climatic conditions during cultivation.

Wines that were produced from Rondo variety contained between 573.25 and 655.12 mg/L of total phenolic compounds. The wines of the Rondo variety had the highest concentrations of anthocyanins, followed by flavan-3-ols, phenolic acids, stilbenes, and flavonols. The subtotal concentration of anthocyanins ranged from 390.91 to 445.76 mg/L, which was mainly due to the concentrations of malvidin 3-*O*-glucoside-5-*O*-glucoside and malvidin 3-*O*-glucoside. The concentration of flavan-3-ols varied from 113.98 to 177.23 mg/L. The flavan-3-ols that were found in appreciable concentrations in the wines were (+) catechin, procyanidin B-type 3 and procyanidin B1. The subtotal concentration of phenolic acids ranged from 32.16 to 45.98 mg/L. This class of phenolic compounds was mainly composed of gallic acid and caftaric acid. No ferulic acid was found in one wine that was produced from Rondo variety, whereas, in the remaining nine wines, the concentrations of this acid were low. The concentration of stilbenes varied from 7.43 to 10.31 mg/L. *Cis*-piceid was the first abundant stilbene and *cis*-resveratrol was the second one. The subtotal content of flavonols ranged from 1.50 to 2.18 mg/L. Isorhamnetin 3-*O*-glucoside and myricetin-3-*O*-glucoside were the main flavonols. In recent years, several papers have been published regarding the content of phenolic compounds in wines that were produced from the Rondo variety. Similar to our studies, Ruocco et al. (2019) [34] and Kapusta et al. (2018) [36] found that anthocyanins constituted the largest class of phenolic compounds in Rondo wines, mainly malvidin 3-*O*-glucoside-5-*O*-glucoside and malvidin 3-*O*-glucoside. Furthermore, catechin was one of the main flavan-3-ols and caftaric acid was one of the main phenolic acids. Myricetin-3-*O*-glucoside was one of the most abundant flavonols in our study, which is in agreement with Kapusta et al. (2018) [36]. On the contrary, Ruocco et al. (2019) [34] and Kapusta et al. (2018) [36] showed low concentrations of isorhamnetin 3-*O*-glucoside. Similarly, *cis*-piceid and *cis*-resveratrol were found in significant amounts in wines that were tested by Kapusta et al. (2018) [36], while Ruocco et al. (2019) [34] determined significant amounts of *cis*-piceid, while not detecting *cis*-resveratrol. In our work, the contents of (+) catechin, (−) epicatechin, gallic acid, caffeic acid, and p-coumaric acid in Rondo wines differed from the contents of these compounds in wine of the same grape variety that was tested by Socha et al. (2015) [40]. Only ferulic acid contents were similar. This could be due to the differences in sample preparation and chromatographic conditions between these studies.

**Figure 4:**
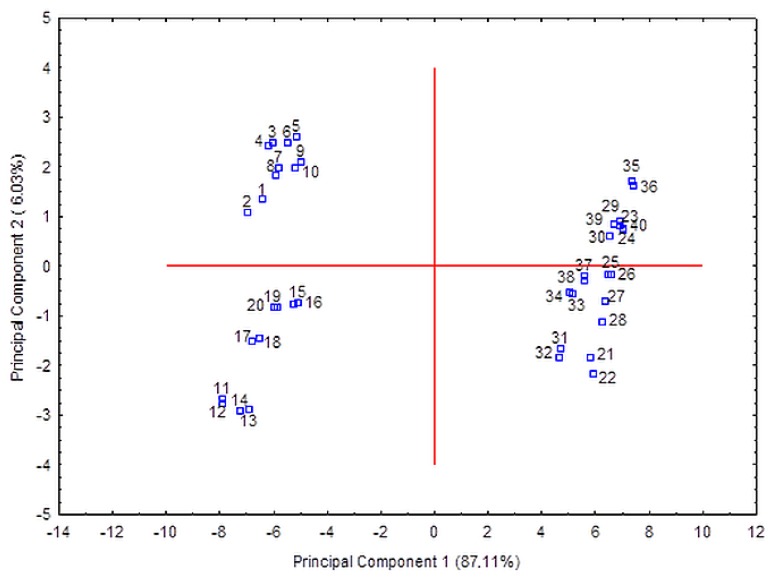
Score plot of wine samples on the PCA plane defined by the first two principal components (R1-R5 wines: 1–10, R1 LAB–R5 LAB wines: 11–20, Z1-Z5 wines: 21–30, Z1 LAB–Z5 LAB wines: 31–40; see Table 1 for phenolic compounds names).

The Student’s t-test and Mann–Whitney test revealed significant differences in the content of anthocyanins, flavonols, and stilbenes among wines the produced from Zweigelt and Rondo varieties. The application of these tests was possible because the Liliefors test used earlier did not reject the hypothesis of normally distributed data. In the case of phenolic acids, only Gal and Prot were significant, while in the flavan-3-ols group, statistical significance was detected in Cat, Epicat, ProcB1, ProcB-type2, ProcB-type3, ProcB-type4, ProcC1, ProcC-type1, ProcC-type 2 (Tables S2 and S3). Then the PCA was performed to the data set by using these 46 significant compounds as variables. PCA revealed that the two first principal components explained 93.14% of the total variance. The first PC accounts for 87.11% and the second PC for 6.03% of the variance. A plot of the scores of PC1 versus PC2 (Figure 4) demonstrated that the wines of Rondo and Zweigelt formed two completely separate groups. Moreover, in the group of Rondo wines, the wines that had undergone spontaneous malolactic fermentation were separated from the wines that had undergone induced malolactic fermentation.

**Figure 5:**
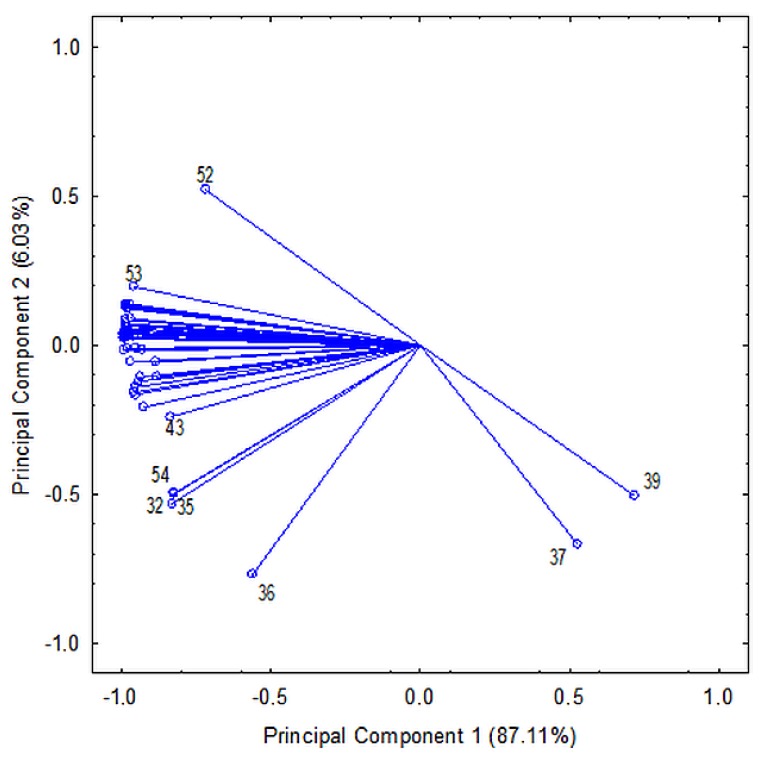
Projection of variables on the PCA plane (in order to improve clarity of the drawing, the following numbers of compounds, which were placed between compound No. 53 and 43, have been omitted: 1–31, 34, 40, 42, 44, 45, and 55; see Table 1 for phenolic compounds names).

**Figure 6:**
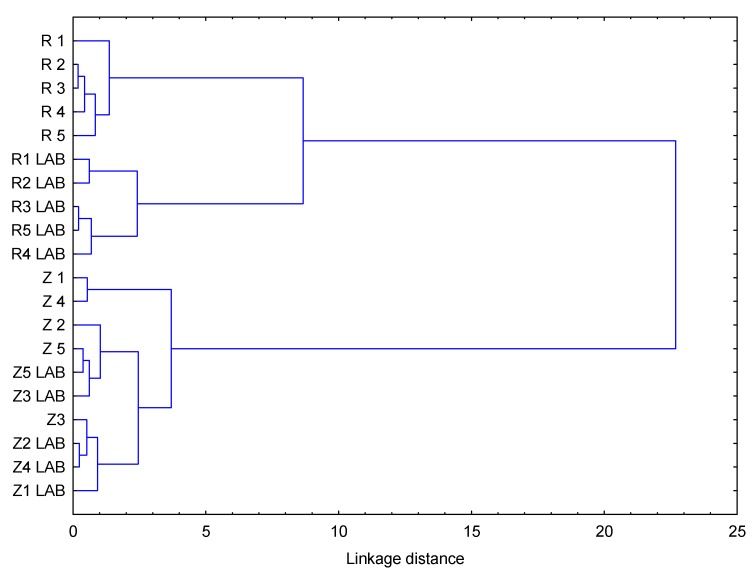
Dendrogram showing clustering of wines made of Zweigelt and Rondo varieties (R1-R5-wines from the Rondo variety, in which alcoholic fermentation was carried out by different yeast strains and malolactic fermentation was spontaneous; R1 LAB–R5 LAB—wines from the Rondo variety, in which alcoholic fermentation was carried out by different yeast strains (but the same as in R1–R5 wines), and malolactic fermentation was induced; Z1-Z5-wines from the Zweigelt variety, in which alcoholic fermentation was carried out by different yeast strains and malolactic fermentation was spontaneous; Z1 LAB–Z5 LAB-wines from the Zweigelt variety, in which alcoholic fermentation was carried out by different yeast strains (but the same as in Z1–Z5 wines), and malolactic fermentation was induced).

After analyzing the basic descriptive statistics and the distance between observations from both groups of wines, the compounds that best differentiated the tested wines were selected: from the stilbenes group—Cis-P and Cis-R, from the flavonols group—3-Giso, 3-RhadQ, and 3GluQ, from the flavan-3-ols group—ProcC1 and ProcC-type2, from the phenolic acids group—Prot and Gal. In the case of anthocyanins, the best differentiating compounds (the largest distance between observations) were 35dGM, 35dGPeo, 3gD, 35dGD, and 35dGPet due to their number and because they all are significant. HCA was performed in order to analyze the similarity between examined wines. Their number had to be reduced due to excess of significant variables. First, principal component analysis (PCA) was performed to show correlations between variables (Figure 5). The majority of them are strongly correlated with each other. While considering results of PCA and HCA, the variables were divided into four groups. The first contained only Trans-P, the second—Epicat and ProcB-type2, the third—Cat, ProcB1, Trans-R, ProcC-type1, and the fourth—all other compounds: 35dGC, 35dGD, 35dGM, 35dGPeo, 35dGPet, 3ag5gPet, 3agC, 3agD, 3agM, 3agPeo, 3agPet, 3cafGD, 3gC, 3gD, 3gM, 3gPeo, 3gPet, 3kg5gM, 3kg5gPeo, 3kgC, 3kgD, 3kgM, 3kgPeo, 3kgPet, 3-Giso, 3-GM, 3-GQ, 3-RhadQ, 3-GluQ, 3-RutM, 3-RutQ, Cis-P, Cis-R, Gal, Prot, ProcB-type3, ProcB-type4, ProcC1, and ProcC-type2. Subsequently, the representative of each of four groups was selected. The representative of the first group was Trans-P variable. PCA was applied for variables from these groups to find the representatives of the other three groups. The first principal component was chosen to be the representative of each group. The reduction of variable numbers did not cause any significant loss of information—a maximum of 6.63% for the fourth most numerous group. The wines’ clustering revealed that wines made from Zweigelt and Rondo grapes formed two separate groups, the Rondo group was divided into two subgroups that differed in the type of malolactic fermentation. The wines that were produced from one grape variety showed greater similarity while taking into account phenolic compounds than those produced from different varieties (Figure 6).

## 3. Materials and Methods

### 3.1. Chemicals 

Sodium hydroxide solution (0.1 mol/L) was purchased from Sigma-Aldrich (Poznań, Poland). Buffer solutions of pH 2, 4 and 7 were purchased from POCH (Gliwice, Poland). Sucrose, glucose, fructose, tartaric, malic, lactic, acetic, citric and succinic acids were obtained from Sigma-Aldrich (Poznań, Poland), all with a purity level of ≥98%. Analytical standards of cyanidin-3*-O-*glucoside, peonidin-3-Oglucoside, petunidin-3*-O-*glucoside, malvidin-3*-O-*glucoside, delphinidin-3*-O-*glucoside, myricetin-3*-O-*glucoside, quercetin-3*-O-*rutinoside, quercetin-4′*-O-*glucoside, quercetin-3*-O-*glucoside, kaempferol-3*-O-*glucoside, isorhamnetin-3*-O-*glucoside, (+)-catechin, (−)-epicatechin, (−)-epicatechin-3-gallate, procyanidin A1 and A2, *trans*-resveratrol, and *trans*-piceid, were purchased from Extrasynthese (Lyon, France). The analytic standards of gallic acid, caftaric acid, protocatechuic acid, coutaric acid, caffeic acid, *p-*coumaric acid, and ferulic acid were purchased from PhytoLab (Vestenbergsgreuth, Germany). Acetonitrile (HPLC gradient grade) was purchased from POCH (Gliwice, Poland). Formic acid (LC–MS grade) was purchased from Fischer Scientific (Schwerte, Germany).

### 3.2. Winemaking and Wine Samples

The grapes of Zweigelt and Rondo varieties originated from ‘Małe Dobre’ and ‘Dom Bliskowice’ vineyards, respectively. The vineyards are located in the Lublin Province, Poland. The harvest was manually done in 2017. The grapes were taken to the laboratory, and then stalked, crushed, portioned, and transferred to 5 L fermentation vessels. The grapes from the Zweigelt variety were transferred to five fermentation vessels, and grapes from the Rondo variety to other five vessels. One of the following commercial *Saccharomyces cerevisiae* preparations: SafŒno ™ SC 22, Essentiale Grand Cru (Lesaffre, France), Siha Active Yeast 8, Siha Rubino Cru (Eaton, Tinton Falls, NJ, USA), and *S. cerevisiae* × *S. bayanus* preparation—SafŒno ™ HD S62 (Lesaffre, France), was added to one fermentation vessel with Zweigelt variety, according to the manufacturer’s instructions. Similarly, the same yeast was added to fermentation vessels with Rondo variety. The fermentation vessels were closed with stoppers and fermentation tubes. The alcoholic fermentations were conducted at 22–24 °C and then monitored by weight loss starting at inoculation and continuing every 24 h until the weight was stabilized. At the end of fermentation, each wine was pressed with a basket press and divided into two vessels. *Oenococcus oeni* preparation—Viniflora Oenos (Eaton, Tinton Falls, NJ, USA) was added to wine in one vessel and malolactic fermentation was carried out (induced malolactic fermentation). The lactic acid bacteria were not added to the wine in the second vessel (spontaneous malolactic fermentation). Table 2 presents a description of wines. Wines in both vessels were decanted, potassium metabisulfite at a dose of 60 mg/L was added, and wines were placed at 15 °C for eight weeks. Subsequently, wines were decanted again and the temperature was lowered to 8 °C for 4 weeks. After this, the wines were decanted, potassium metabisulfite at a dose of 60 mg/L was added, and the wines were bottled and stored at 15 °C until analysis.

### 3.3. Determination of Oenological Parameters

The total acidity was determined according to OIV-MA-AS313-01 method while using potentiometric titration and expressed as g of tartaric acid in L [47] and the pH was determined according to OIV-MA-AS313-15 [48].

A HPLC system (Shimadzu, Kyoto, Japan) that consisted of a DGU-20A3 mobile phase degasser, two LC-20AD pumps, a CBM20A communication module, a SIL-20ACHT autosampler, a CTO-20AC column oven, a RID-10A refractive index detector, SPD-M20A photodiode array detector, and LCsolution program was used for the analysis of sugars and organic acids in wine samples. The wine samples before injection were degassed in an ultrasonic water bath, and then filtered through a 0.45 µm SimplePure PTFE syringe filters (Membrane Solutions, Auburn, WA, USA) and diluted with deionized water at 1:4 ratio (*v*/*v*). The separation of sugars was performed with isocratic elution on a cation exchange chromatography column Rezex RCM-Monosacharide Ca++ (300 mm × 7.8 mm, 5 μm particle size). Mobile phase was EDTA calcium disodium salt at a concentration of 0.1 mmol/L in deionized water, at a flow rate of 0.6 mL/min. The temperature of the column was maintained at 70 °C. The volume of injected sample was 5 μL and the time of analysis was 25 min. A refractive index detector was used for the identification and quantification of sugars. The separation of organic acids was achieved in isocratic mode with a mobile phase of phosphate solution in deionized water containing 50 mM H_3_PO_4_ and 10 mM NaH_2_PO_4_ at pH 1.9. The flow rate was 0.6 mL/min. The Luna 5u C18 (2) Phenomenex chromatographic column (250 mm × 4.6 mm, 5 μm particle size), which was set at 20 °C, was used. The volume of injected sample was 20 μL and time of analysis was 30 min. The identification and quantification of organic acids were done using photodiode array detector at 214 nm. Stock standard solutions of 10 g/L for sugars and 8 g/L for organic acids were prepared by dissolving each sugar and organic acid in deionized water. The working standard solutions were prepared by dilution with deionized water. The quantification was carried out using the external standard method. All of the determinations were performed in duplicate and expressed as g/L.

### 3.4. Determination of Polyphenolic Compounds

The analytical procedure was performed applying the method that was previously described by Kapusta et al. (2018) [36]. Polyphenolic compounds were analyzed while using ultra-performance reverse-phase liquid chromatography UPLC-PDA-MS/MS Waters ACQUITY system (Waters, Milford, MA, USA) consisting of a binary pump manager, sample manager, column manager, photodiode array (PDA) detector, and tandem quadrupole mass spectrometer (TQD) with electrospray ionization (ESI). The separation was carried out using a BEH C18 column (100 mm × 2.1 mm i.d., 1.7 µm, Waters) that was kept at 50 °C. For the anthocyanins investigation, the following solvent system was applied: mobile phase A (2% formic acid in water, *v*/*v*) and mobile phase B (2% formic acid in 40% ACN in water, *v*/*v*). For other polyphenolic compounds, a lower concentration of formic acid was used (0.1%, *v*/*v*). The gradient program was set, as follows: 0 min. 5% B, from 0 to 8 min. linear to 100% B, and from 8 to 9.5 min. for washing and back to initial conditions. The injection volume of samples was 5 µL (partial loop with needle overfill), and the flow rate was 0.35 mL/min. The following parameters were used for TQD: capillary voltage 3.5 kV, con voltage, 30 V in positive and negative mode; the source was kept at 120 °C and the desolvation temperature was 350 °C, con gas flow 100 L/h, and desolvation gas flow 800 L/h. Argon was used as the collision gas at a flow rate of 0.3 mL/min. The polyphenolic detection and identification were based on specific PDA spectra, mass-to-charge ratio, and fragment ions obtained after collision-induced dissociation (CID). Before injection, the wine samples were filtered through a 0.45-µm Millipore filter and then directly injected onto the chromatographic column. Quantification was achieved by the injection of solutions of known concentrations that ranged from 0.05 to 5 mg/mL (R^2^ ≤ 0.9998) of phenolic compounds as standards. All of the determinations were performed in duplicate and expressed as mg/L. Waters MassLynx software v.4.1 was used for data acquisition and processing.

The method was validated for parameters, such as: linearity, accuracy (relative error, RE), limit of detection (LOD), limit of quantification (LOQ), and precision (relative standard deviation, RSD). The stock solutions of five polyphenols were prepared by dissolving standards in methanol, followed by dilution to final solutions. The concentrations against peak area were plotted. The regression equation was obtained by the weighted least-squares linear regression. The LOD was determined as a signal-to-noise ratio (S/N) of 3:1, and the LOQ was determined as a S/N of 10:1. An acceptable RE within ±20% and an RSD within 80–120% were obtained.

### 3.5. Chemometric Analysis

Data analysis was conducted using the Statistica 13.1 software package (StatSoft, Krakow, Poland). The Lilliefors test was carried out to examine whether the data come from the normally distributed population. The Student’s t-test was used to examine whether there were any statistically significant differences between wines that were made from Zweigelt and Rondo varieties and whenever the Student’s t-test assumptions regarding the homogeneity of variance were not fulfilled, the Mann–Whitney non-parametric test was applied. PCA was used to visualize eventual grouping of samples and also show correlations between variables. HCA was applied to explore similarity between tested wines. Clustering was performed by means of the Ward distance matrix that formed on the basis of the Euclidean distance.

## 4. Conclusions

Red wines that were produced from the Zweigelt (*V. vinifera*) variety and Rondo (non-*V. vinifera*) variety were characterized in this paper. For the first time, the oenological parameters and the profile of phenolic compounds in Zweigelt wines were presented.

Phenolic compounds were determined while using the UPLC-PDA-MS/MS method. Zweigelt wines had high concentrations of anthocyanin monoglucosides, such as malvidin 3*-O-*glucoside, delphinidin 3*-O-*glucoside, and petunidin 3*-O-*glucoside, while the Rondo wines had high concentrations of malvidin 3*-O-*glucoside-5*-O-*glucoside, peonidin 3*-O-*glucoside-5*-O-*glucoside, and delphinidin 3*-O-*glucoside-5*-O-*glucoside. Wines that were made from the Rondo variety contained higher amounts of gallic acid than Zweigelt wines. Red wines of both varieties mainly contained isorhamnetin 3*-O-*glucoside, and myricetin-3*-O-*glucoside. The fraction of flavan-3-ols mainly composed of (−) epicatechin and (+) catechin in Zweigelt wines and of (+) catechin, procyanidin B-type 3 and procyanidin B1 in Rondo wines. Rondo and Zweigelt wines differed in the content of individual stilbenes, especially *trans*-piceid and *cis-*piceid.

HCA revealed that wines that were made from Zweigelt and Rondo grapes formed two separate groups; the Rondo group was divided into two subgroups differing in the type of malolactic fermentation. Such results enhance the potential of HCA in detecting wine adulteration.

The determination of phenolic compounds using UPLC-PDA-MS/MS and HCA allowed for the classification of Zweigelt and Rondo wines, regardless of yeast strain and type of malolactic fermentation (spontaneous or induced). To date, no work has been published regarding the possibility of varietal classification of red wines made in Poland. Further research is needed on wines that are produced from Zweigelt and Rondo varieties that originated from different regions of Poland to show whether the wines can be classified independently of the region of origin.

## Figures and Tables

**Table 1:** UPLC-PDA-MS/MS identification parameters of phenolic compounds.

No	Compound	Abbreviation	RT (min.)	[M − H] (*m*/*z*)	Fragment ions (*m*/*z*)	λmax (nm)
Anthocyanins						
1	Delphinidin 3-*O*-glucoside-5-*O*-glucoside	35dGD	2.04	627	465, 303	277, 525
2	Cyanidin 3-*O*-glucoside-5-*O*-glucoside	35dGC	2.19	611	449, 287	280, 516
3	Delphinidin 3-*O*-glucoside	3gD	2.38	465	303	280, 523
4	Petunidin 3-*O*-glucoside-5-*O*-glucoside	35dGPet	2.53	641	479, 317	277, 531
5	Peonidin 3-*O*-glucoside-5-*O*-glucoside	35dGPeo	2.67	625	463, 301	278, 513
6	Malvidin 3-*O*-glucoside-5-*O*-glucoside	35dGM	2.72	655	493, 331	275, 524
7	Cyanidin 3-*O*-glucoside	3gC	2.74	449	287	279, 515
8	Petunidin 3-*O*-glucoside	3gPet	2.92	479	317	277, 526
9	Peonidin 3-*O*-glucoside	3gPeo	3.31	463	301	279, 515
10	Malvidin 3-*O*-glucoside	3gM	3.43	493	331	278, 530
11	Delphinidin 3-*O*-(6′′-*O*-acetyl)-glucoside	3agD	3.53	507	465, 303	280, 528
12	Cyanidin 3-*O*-(6′′-*O*-acetyl)-glucoside	3agC	3.95	491	449, 287	283, 522
13	Petunidin 3-*O*-(6′′-*O*-acetyl)-glucoside	3agPet	4.10	521	317	280, 530
14	Petunidin 3-*O*-(6′′-*O*-acetyl)-glucoside-5-*O*-glucoside	3ag5gPet	4.28	787	625, 479, 317	280, 530
15	Delphinidin 3-*O*-(6′′-*O*-coumaryl)-glucoside	3kgD	4.47	611	303	279, 530
16	Malvidin 3-*O*-(6′′-*O*-acetyl)-glucoside	3agM	4.62	535	331	280, 521
17	Malvidin 3-*O*-(6′′-*O*-coumaryl)-glucoside-5-*O*-glucoside	3kg5gM	4.67	801	639, 493, 331	280, 530
18	Peonidin 3-*O*-(6′′-*O*-coumaryl)-glucoside-5-*O*-glucoside	3kg5gPeo	4.68	771	609, 463, 301	279, 523
19	Peonidin 3-*O*-(6′′-*O*-acetyl)-glucoside	3agPeo	4.85	505	463, 301	277, 535
20	Cyanidin 3-*O*-(6′′-*O*-coumaryl)-glucoside	3kgC	4.93	595	287	283, 522
21	Petunidin 3-*O*-(6′′-*O*-coumaryl)-glucoside	3kgPet	4.98	625	317	280, 531
22	Delphinidin 3-*O*-(6′′-caffeoyl)-glucoside	3cafGD	5.35	627	465, 303	280, 528
23	Peonidin 3-*O*-(6′′-*O*-coumaryl)-glucoside	3kgPeo	5.39	609	301	279, 523
24	Malvidin 3-*O*-(6′′-*O*-coumaryl)-glucoside	3kgM	5.44	639	331	280, 521
Flavonols						
25	Myricetin-3-*O*-rutinoside	3-RutM	4.08	625	479, 317	255, 353
26	Myricetin-3-*O*-glucoside	3-GM	4.24	479	317	255, 353
27	Quercetin 3-*O*-glucuronide	3-GluQ	4.48	477	301	255, 356
28	Isorhamnetin 3-*O*-glucoside	3-Giso	4.67	447	315	254, 369
29	Quercetin 3-*O*-glucoside	3-GQ	4.82	463	301	253, 365
30	Quercetin 3-*O*-rutinoside	3-RutQ	4.99	609	447, 301	255, 355
31	Dihydroquercetin 3-*O*-ramnoside	3-RhadQ	5.57	449	303	253, 372
Flavan-3-ols						
32	Procyanidin B1	ProcB1	2.66	577	425, 285	280
33	Procyanidin B-type 1	ProcB-type1	2.81	577	425, 285	276
34	Procyanidin C1	ProcC1	2.97	865	577, 285	280
35	(+) catechin	Cat	3.01	289	-	280
36	Procyanidin C-type 1	ProcC-type1	3.07	865	577, 285	280
37	Procyanidin B-type 2	ProcB-type2	3.31	577	285	279
38	Procyanidin B2	ProcB2	3.34	577	285	280
39	(−) epicatechin	Epicat	3.68	289	-	280
40	Procyanidin C-type 2	ProcC-type2	3.74	865	577, 289	279
41	Procyanidin C-type 3	ProcC-type3	3.84	865	577, 289	280
42	Procyanidin B-type 3	ProcB-type3	4.06	577	289	279
43	Procyanidin B-type 4	ProcB-type4	4.34	577	289	280
Phenolic acids						
44	Gallic acid	Gal	1.47	169	125	272
45	Protocatechuic acid	Prot	2.25	153	109	308
46	Caftaric acid	Caft	2.49	311	179	328, 294
47	Coutaric acid	Cout	3.08	295	163	310
48	Caffeic acid	Caff	3.46	153	109	260, 294
49	Ferulic acid	Fer	4.92	193	134	323, 293
50	*p-*Coumaric acid	*p-*Coum	4.39	163	119	308
51	Coumaric acid	Coum	4.82	163	119	310
Stilbenes						
52	*Trans*-piceid	*Trans*-P	4.75	389	227	327
53	*Cis-*piceid	*Cis-*P	5.94	389	227	327
54	*Trans*-resveratrol	*Trans*-R	6.24	227	185	327
55	*Cis-*resveratrol	*Cis-*R	7.42	227	143	327

*m*/*z* for anthocyanins have been obtained in the positive mode ([M + H]^+.^

**Table 2:** Description of wine samples.

Wine Code	Grape Variety	Yeast	Lactic Acid Bacteria
Z1	Zweigelt	SafŒno ™ SC 22	-
Z1 LAB	Zweigelt	SafŒno ™ SC 22	Viniflora Oenos
Z2	Zweigelt	SafŒno ™ HD S62	-
Z2 LAB	Zweigelt	SafŒno ™ HD S62	Viniflora Oenos
Z3	Zweigelt	Essentiale Grand Cru	-
Z3 LAB	Zweigelt	Essentiale Grand Cru	Viniflora Oenos
Z4	Zweigelt	Siha Active Yeast 8	-
Z4 LAB	Zweigelt	Siha Active Yeast 8	Viniflora Oenos
Z5	Zweigelt	Siha Rubino Cru	-
Z5 LAB	Zweigelt	Siha Rubino Cru	Viniflora Oenos
R1	Rondo	SafŒno ™ SC 22	-
R1 LAB	Rondo	SafŒno ™ SC 22	Viniflora Oenos
R2	Rondo	SafŒno ™ HD S62	-
R2 LAB	Rondo	SafŒno ™ HD S62	Viniflora Oenos
R3	Rondo	Essentiale Grand Cru	-
R3 LAB	Rondo	Essentiale Grand Cru	Viniflora Oenos
R4	Rondo	Siha Active Yeast 8	-
R4 LAB	Rondo	Siha Active Yeast 8	Viniflora Oenos
R5	Rondo	Siha Rubino Cru	-
R5 LAB	Rondo	Siha Rubino Cru	Viniflora Oenos

## Supplementary Materials

The following are available on line: Table S1: Oenological parameters of analyzed wines, Table S2: Concentration of phenolic compounds in wines produced from Zweigelt variety (in mg/L), Table S3: Concentration of phenolic compounds in wines produced from Rondo variety (in mg/L), Figure S1: Chromatogram of sugars in Rondo wine, Figure S2: Chromatogram of sugars in Zweigelt wine, Figure S3: Chromatogram of sugars standards, Figure S4: Chromatogram of organic acids in Rondo wine, Figure S5: Chromatogram of organic acids in Zweigelt wine, Figure S6: Chromatogram of organic acid standards, Figure S7: Single ion recording (SIR) of anthocyanins in Rondo wine detected by UPLC-MS/MS (part 1), Figure S8: Single ion recording (SIR) of anthocyanins in Rondo wine detected by UPLC-MS/MS (part 2), Figure S9: Single ion recording (SIR) of flavonols in Rondo wine detected by UPLC-MS/MS, Figure S10: Single ion recording (SIR) of flavan-3-ols in Rondo wine detected by UPLC-MS/MS, Figure S11: Single ion recording (SIR) phenolic acids in Rondo wine detected by UPLC-MS/MS, Figure S12: Single ion recording (SIR) of stilbenes in Rondo wine detected by UPLC-MS/MS.
