# Supplementary material for: Classification of Red Wines Produced from Zweigelt and Rondo Grape Varieties Based on the Analysis of Phenolic Compounds by UPLC-PDA-MS/MS
Source: Molecules. 2020 Mar 16;25(6):1342. doi: 10.3390/molecules25061342 (PMC7145314; doi:10.3390/molecules25061342)
Supplement: Supplementary file 1 [file molecules-25-01342-s001.pdf]

Classification of Red Wines Produced from Zweigelt and Rondo Grape Varieties Based on the Analysis of Phenolic Compounds by UPLC-PDA-MS/MS

Anna Stój [1,*], Ireneusz Kapusta [2] and Dorota Domagała [3]

[1] Department of Biotechnology, Microbiology and Human Nutrition, Faculty of Food Science and Biotechnology, University of Life Sciences in Lublin, 8 Skromna Street, 20-704 Lublin, Poland

[2] Department of Food Technology and Human Nutrition, College of Natural Science, Rzeszów University, 4 Zelwerowicza Street, 35-601 Rzeszów, Poland

[3] Department of Applied Mathematics and Computer Science, Faculty of Production Engineering, University of Life Sciences in Lublin, 28 Głęboka Street, 20-612 Lublin, Poland

* Correspondence: anna.stoj@up.lublin.pl; Tel.: +48-81-4623380

Table S1. Oenological parameters of analyzed wines

	Wines																			
	R1	R2	R3	R4	R5	R1 LAB	R2 LAB	R3 LAB	R4 LAB	R5 LAB	Z1	Z2	Z3	Z4	Z5	Z1 LAB	Z2 LAB	Z3 LAB	Z4 LAB	Z5 LAB
Sugars (g/L)																				
Sucrose	0.49	0.32	0.26	0.43	0.27	6.84	5.87	6.00	6.00	3.07	0.71	1.02	2.66	nd	0.51	3.12	3.00	2.71	2.59	3.45
Glucose	nd	nd	nd	nd	nd	nd	nd	nd	nd	nd	0.80	0.81	0.73	0.78	0.84	0.80	0.83	0.79	0.84	0.83
Fructose	1.31	1.27	1.28	1.27	1.57	1.55	1.41	1.18	1.14	1.17	1.67	1.59	1.61	1.61	1.57	1.77	1.51	1.58	1.34	1.61
Acids (g/L)																				
Tartaric acid	1.19	2.63	1.65	2.26	2.07	1.83	2.14	1.76	1.94	3.60	3.91	2.54	3.57	3.19	3.37	2.76	3.31	3.04	2.53	3.57
Malic acid	13.44	13.60	11.60	10.40	12.10	8.55	11.72	9.57	8.59	9.89	7.00	8.40	6.53	6.32	7.62	5.63	6.34	5.87	4.86	5.82
Lactic acid	1.24	1.23	1.35	1.48	1.15	5.38	4.66	4.86	4.78	2.73	0.96	1.22	2.62	1.07	1.16	2.75	2.89	2.53	2.44	2.75
Acetic acid	0.58	0.64	0.74	0.61	0.74	0.93	1.00	1.01	0.97	0.79	0.64	0.63	0.90	0.88	1.30	0.76	0.81	0.74	0.90	0.81
Citric acid	0.65	0.65	0.75	0.63	0.67	0.41	0.42	0.47	0.35	0.38	0.53	0.49	0.31	0.40	0.44	0.27	0.28	0.30	0.21	0.27
Succinic acid	0.17	0.14	0.11	0.14	0.14	nd	nd	nd	nd	nd	0.06	0.08	nd	0.06	0.06	nd	nd	nd	nd	nd
pH	3.66	3.71	3.67	3.72	3.63	3.77	3.78	3.79	3.81	3.77	3.35	3.38	3.49	3.40	3.37	3.02	3.50	3.51	3.52	3.51
Total acidity (g/L)	6.34	6.19	6.11	5.78	5.77	5.18	4.95	4.58	4.20	4.24	5.40	5.40	4.50	4.80	5.33	5.17	5.02	4.65	3.94	4.76

R1-R5 wines from the Rondo variety, in which alcoholic fermentation was carried out by different yeast strains and malolactic fermentation was spontaneous; R1 LAB-R5 LAB wines from the Rondo variety, in which alcoholic fermentation was carried out by different yeast strains (but the same as in R1-R5 wines), and malolactic fermentation was induced; Z1-Z5 wines from the Zweigelt variety, in which alcoholic fermentation was carried out by different yeast strains and malolactic fermentation was spontaneous; Z1 LAB-Z5 LAB wines from the Zweigelt variety, in which alcoholic fermentation was carried out by different yeast strains (but the same as in Z1-Z5 wines), and malolactic fermentation was induced

Table S2. Concentration of phenolic compounds in wines produced from Zweigelt variety (in mg/L)

		Wines												
		Z1	Z2	Z3	Z4	Z5	Mean ± ΔS[a]	Z1 LAB	Z2 LAB	Z3 LAB	Z4 LAB	Z5 LAB	Mean ± ΔS[b]	Mean ± ΔS[c]
Anthocyanins														
1	Delphinidin 3-*O*-glucoside-5-*O*-glucoside	0.86	0.85	0.86	0.86	0.85	0.85 *± 0.00	0.85	0.88	0.86	0.88	0.85	0.86 *± 0.01	0.86* ± 0.01
2	Cyanidin 3-*O*-glucoside-5-*O*-glucoside	0.22	0.21	0.22	0.21	0.21	0.21 *± 0.00	0.20	0.21	0.19	0.22	0.21	0.20 *± 0.01	0.21* ± 0.01
3	Delphinidin 3-*O*-glucoside	7.71	2.49	5.56	4.92	4.88	5.11 *± 1.76	12.28	9.12	3.06	9.10	3.29	7.37 *± 3.81	6.24* ± 3.12
4	Petunidin 3-*O*-glucoside-5-*O*-glucoside	0.25	0.23	0.24	0.24	0.25	0.24 *± 0.00	0.26	0.25	0.23	0.25	0.24	0.24 *± 0.01	0.24* ± 0.01
5	Peonidin 3-*O*-glucoside-5-*O*-glucoside	0.65	0.62	0.62	0.62	0.72	0.64 *± 0.04	0.62	0.62	0.61	0.63	0.66	0.62 *± 0.01	0.64* ± 0.03
6	Malvidin 3-*O*-glucoside-5-*O*-glucoside	0.38	0.41	0.31	0.39	0.21	0.33 *± 0.08	0.37	0.33	0.30	0.30	0.28	0.31 *± 0.03	0.33* ± 0.06
7	Cyanidin 3-*O*-glucoside	0.56	0.34	0.59	0.50	0.50	0.50 *± 0.09	0.81	0.76	0.51	0.75	0.44	0.65 *± 0.15	0.58* ± 0.15
8	Petunidin 3-*O*-glucoside	3.84	0.96	2.67	2.19	2.53	2.43 *± 0.97	6.60	4.53	1.37	4.48	1.74	3.74 *± 2.05	3.09* ± 1.70
9	Peonidin 3-*O*-glucoside	1.15	0.68	1.10	0.99	1.05	0.99 *± 0.17	1.79	1.62	0.79	1.54	0.96	1.33 *± 0.41	1.17* ± 0.36
10	Malvidin 3-*O*-glucoside	42.00	8.65	36.51	24.78	32.43	28.87 *± 12.20	76.84	57.80	17.86	56.15	28.58	47.44 *± 22.51	38.16* ± 20.04
11	Delphinidin 3-*O*-(6′′-*O*-acetyl)-glucoside	1.41	0.93	1.21	1.12	1.23	1.17 *± 0.16	2.05	1.58	1.00	1.55	1.08	1.45 *± 0.40	1.31* ± 0.33
12	Cyanidin 3-*O*-(6′′-*O*-acetyl)-glucoside	0.18	0.18	0.18	0.18	0.18	0.17 *± 0.00	0.18	0.18	0.18	0.18	0.18	0.17 *± 0.00	0.18* ± 0.00
13	Petunidin 3-*O*-(6′′-*O*-acetyl)-glucoside	0.37	0.25	0.34	0.30	0.31	0.31 *± 0.04	0.52	0.43	0.28	0.41	0.29	0.38 *± 0.09	0.35* ± 0.08
14	Petunidin 3-*O*-(6′′-*O*-acetyl)-glucoside-5-*O*-glucoside	0.23	0.23	0.23	0.23	0.23	0.22 *± 0.00	0.23	0.23	0.23	0.23	0.23	0.22 *± 0.00	0.23* ± 0.00
15	Delphinidin 3-*O*-(6′′-*O*-coumaryl)-glucoside	1.00	0.89	0.94	0.90	0.98	0.94 *± 0.04	1.13	1.02	0.89	0.99	0.93	0.99 *± 0.08	0.97* ± 0.07
16	Malvidin 3-*O*-(6′′-*O*-acetyl)-glucoside	2.05	0.20	1.97	0.79	1.54	1.31 *± 0.75	5.47	3.49	0.61	3.40	1.22	2.83 *± 1.84	2.07* ± 1.58
17	Malvidin 3-*O*-(6′′-*O*-coumaryl)-glucoside-5-*O*-glucoside	0.33	0.47	0.39	0.40	0.40	0.39 *± 0.04	0.28	0.34	0.45	0.31	0.42	0.35 *± 0.06	0.38* ± 0.06
18	Peonidin 3-*O*-(6′′-*O*-coumaryl)-glucoside-5-*O*-glucoside	0.58	0.58	0.58	0.58	0.58	0.58 *± 0.00	0.58	0.58	0.58	0.58	0.58	0.57 *± 0.00	0.58* ± 0.00
19	Peonidin 3-*O*-(6′′-*O*-acetyl)-glucoside	0.87	0.64	0.84	0.73	0.82	0.77 *± 0.08	1.22	1.03	0.69	0.98	0.79	0.94 *± 0.19	0.86* ± 0.17
20	Cyanidin 3-*O*-(6′′-*O*-coumaryl)-glucoside	0.51	0.52	0.42	0.54	0.23	0.44 *± 0.19	0.69	0.54	0.22	0.57	0.38	0.47 *± 0.18	0.46* ± 0.19
21	Petunidin 3-*O*-(6′′-*O*-coumaryl)-glucoside	0.31	0.24	0.28	0.26	0.29	0.27 *± 0.02	0.41	0.35	0.25	0.32	0.26	0.31 *± 0.06	0.30* ± 0.05
22	Delphinidin 3-*O*-(6′′-caffeoyl)-glucoside	0.87	0.88	0.87	0.85	0.88	0.86 *± 0.01	0.91	0.90	0.89	0.90	0.87	0.89 *± 0.01	0.88* ± 0.02
23	Peonidin 3-*O*-(6′′-*O*-coumaryl)-glucoside	0.76	0.61	0.73	0.65	0.72	0.69 *± 0.05	1.04	0.91	0.64	0.82	0.68	0.81 *± 0.15	0.76* ± 0.13
24	Malvidin 3-*O*-(6′′-*O*-coumaryl)-glucoside	1.49	0.26	1.42	0.29	1.24	0.93 *± 0.58	4.73	3.17	0.08	2.35	0.74	2.21 *± 1.77	1.58* ± 1.44
Subtotal		68.58	22.34	59.08	43.51	53.28		120.05	90.89	32.75	87.88	45.88		

Table S2 *Cont.*

		Wines												
		**Z1**	**Z2**	**Z3**	**Z4**	**Z5**	**Mean ± ΔS[a]**	**Z1 LAB**	**Z2 LAB**	**Z3 LAB**	**Z4 LAB**	**Z5 LAB**	**Mean ± ΔS[b]**	**Mean ± ΔS[c]**
Flavonols														
25	Myricetin-3-*O*-rutinoside	0.02	0.02	0.01	0.02	0.02	0.01 *± 0.00	0.03	0.02	0.02	0.03	0.01	0.02 *± 0.00	0.02* ± 0.01
26	Myricetin-3-*O*-glucoside	0.28	0.21	0.13	0.20	0.11	0.18 *± 0.06	0.28	0.28	0.13	0.20	0.10	0.19 *± 0.08	0.19* ± 0.07
27	Quercetin 3-*O*-glucuronide	0.00	0.00	0.00	0.00	0.00	0.00 *± 0.00	0.00	0.00	0.00	0.00	0.00	0.00 *± 0.00	0.00* ± 0.00
28	Isorhamnetin 3-*O*-glucoside	0.30	0.24	0.21	0.26	0.18	0.23 *± 0.04	0.33	0.35	0.20	0.29	0.21	0.27 *± 0.06	0.26* ± 0.06
29	Quercetin 3-*O*-glucoside	0.03	0.02	0.02	0.02	0.02	0.02 *± 0.00	0.03	0.03	0.02	0.03	0.02	0.02 *± 0.00	0.02* ± 0.01
30	Quercetin 3-*O*-rutinoside	0.00	0.00	0.00	0.00	0.00	0.00 *± 0.00	0.01	0.01	0.00	0.00	0.00	0.00 *± 0.00	0.00* ± 0.00
31	Dihydroquercetin 3-*O*-ramnoside	0.00	0.00	0.00	0.00	0.00	0.00 *± 0.00	0.00	0.00	0.00	0.00	0.00	0.00 *± 0.00	0.00* ± 0.00
Subtotal		0.62	0.50	0.38	0.52	0.32		0.68	0.70	0.36	0.56	0.35		
Flavan-3-ols														
32	Procyanidin B1	12.21	7.31	13.24	10.41	9.10	10.45 *± 2.25	17.25	16.45	8.71	14.97	12.94	14.06 *± 3.22	12.26*± 3.28
33	Procyanidin B-type 1	5.69	3.34	3.57	4.68	3.37	4.12 ± 0.97	6.54	5.14	2.41	4.76	3.08	4.38 ± 1.56	4.26 ± 1.27
34	Procyanidin C1	2.56	1.16	1.64	1.96	1.63	1.78 *± 0.49	2.57	2.17	0.93	1.80	1.27	1.75 *± 0.62	1.77* ± 0.55
35	(+) catechin	28.86	20.64	24.80	28.37	20.31	24.59 *± 3.93	27.71	23.49	16.41	23.96	20.11	22.33 *± 4.06	23.47* ± 4.06
36	Procyanidin C-type 1	0.85	0.43	0.47	0.62	0.48	0.56 ± 0.16	0.90	0.72	0.40	0.67	0.47	0.63 *± 0.18	0.60* ± 0.18
37	Procyanidin B-type 2	3.21	1.94	2.14	2.64	1.86	2.35 *± 0.53	2.77	2.04	1.25	2.00	1.69	1.94 ± 0.52	2.15* ± 0.56
38	Procyanidin B2	21.48	12.86	14.69	18.30	14.43	16.35 ± 3.33	18.56	16.24	10.29	15.62	11.49	14.44 ± 3.26	15.4 ± 3.36
39	(-) epicatechin	33.90	24.96	26.44	31.34	21.77	27.68 *± 4.78	27.85	23.19	19.37	24.67	20.49	23.11 *± 3.23	25.4* ± 4.61
40	Procyanidin C-type 2	0.65	0.33	0.49	0.48	0.44	0.47 *± 0.11	0.52	0.58	0.29	0.44	0.32	0.43 *± 0.11	0.45* ± 0.11
41	Procyanidin C-type 3	5.24	2.20	3.02	3.77	2.80	3.40 ± 1.11	5.21	4.06	1.87	3.77	2.34	3.45 ± 1.27	3.43 ± 1.17
42	Procyanidin B-type 3	15.58	11.30	11.41	19.57	17.92	15.15 *± 3.54	10.63	12.75	5.87	11.65	10.25	10.22 *± 2.47	12.69* ± 3.91
43	Procyanidin B-type 4	2.14	1.31	1.57	1.82	1.53	1.67 *± 0.30	2.04	1.59	0.82	1.37	1.05	1.37 *± 0.45	1.52* ± 0.41
Subtotal		132.38	87.77	103.48	123.97	95.65		122.55	108.42	68.62	105.69	85.50		
Phenolic acids														
44	Gallic acid	13.85	13.59	12.92	14.04	14.10	13.69 *± 0.72	12.73	12.18	9.74	11.59	12.58	11.76 *± 1.18	12.73* ± 1.38
45	Protocatechuic acid	0.38	0.46	0.38	0.47	0.51	0.43 *± 0.06	0.41	0.47	0.35	0.46	0.48	0.43 *± 0.05	0.44* ± 0.05
46	Caftaric acid	11.86	13.40	12.31	13.80	12.41	12.75 *± 0.96	9.01	9.37	8.10	8.18	8.42	8.61 *± 0.55	10.68 ± 2.26
47	Coutaric acid	2.85	3.04	3.44	3.36	2.83	3.10 *± 0.28	1.62	1.86	1.46	1.51	1.53	1.59 *± 0.15	2.35 ± 0.80
48	Caffeic acid	0.39	0.38	0.49	0.53	0.49	0.45 *± 0.06	2.54	2.34	2.80	2.41	2.85	2.59 *± 0.22	1.52 ± 1.11

Table S2 *Cont.*

		Wines												
		**Z1**	**Z2**	**Z3**	**Z4**	**Z5**	**Mean ± ΔS[a]**	**Z1 LAB**	**Z2 LAB**	**Z3 LAB**	**Z4 LAB**	**Z5 LAB**	**Mean ± ΔS[b]**	**Mean ± ΔS[c]**
49	Ferulic acid	0.07	0.07	0.04	0.05	0.04	0.05 ± 0.01	0.06	0.06	0.02	0.03	0.02	0.03 *± 0.01	0.05 ± 0.02
50	p-Coumaric acid	0.35	0.43	1.10	0.89	1.00	0.75 ± 0.31	0.88	0.83	1.45	1.30	1.49	1.19 *± 0.29	0.97 ± 0.38
51	Coumaric acid	0.11	0.11	0.13	0.16	0.13	0.12 *± 0.01	0.24	0.21	0.27	0.28	0.28	0.25 *± 0.03	0.19 ± 0.07
Subtotal		29.86	31.47	30.79	33.29	31.52		27.50	27.31	24.20	25.75	27.66		
Stilbenes														
52	Trans-piceid	0.21	0.23	0.08	0.27	0.14	0.18 *± 0.07	0.06	0.10	0.04	0.09	0.05	0.06 *± 0.02	0.13* ± 0.08
53	Cis-piceid	0.96	1.19	0.34	1.36	0.74	0.91 *± 0.37	0.53	0.88	0.28	0.74	0.44	0.57 *± 0.22	0.75* ± 0.35
54	Trans-resveratrol	0.25	0.18	0.18	0.21	0.14	0.19 *± 0.03	0.30	0.31	0.19	0.25	0.15	0.23 *± 0.06	0.22* ± 0.06
55	Cis-resveratrol	1.65	1.50	1.59	1.58	1.20	1.50 *± 0.17	1.94	1.99	1.58	1.79	1.49	1.75 *± 0.20	1.63* ± 0.23
Subtotal		3.07	3.10	2.19	3.43	2.23		2.84	3.28	2.09	2.87	2.14		
Total		234.51	145.19	195.93	204.71	182.99		273.62	230.60	128.02	222.75	161.53		

Z1-Z5-wines from the Zwigelt variety, in which alcoholic fermentation was carried out by different yeast strains and malolactic fermentation was spontaneous; Z1 LAB-Z5 LAB-wines from the Zweigelt variety, in which alcoholic fermentation was carried out by different yeast strains (but the same as in Z1-Z5 wines), and malolactic fermentation was induced

a - (*) in the same row of Table S2 and Table S3 indicates significant difference between the means of Z1-Z5 and R1-R5 wines ($p < 0.0001$)

b - (*) in the same row of Table S2 and Table S3 indicates significant difference between the means of Z1 LAB-Z5 LAB and R1 LAB-R5 LAB wines ($p < 0.0001$)

c – (*) in the same row of Table S2 and Table S3 indicates mean significantly different among the wines from two selected grape varieties with $p < 0.000001$ (only for procyaninin C-type 1. $p = 0.0337$)

Table S3. Concentration of phenolic compounds in wines produced from Rondo variety (in mg/L)

		Wines												
		**R1**	**R2**	**R3**	**R4**	**R5**	**Mean ± ΔS[a]**	**R1 LAB**	**R2 LAB**	**R3 LAB**	**R4 LAB**	**R5 LAB**	**Mean ± ΔS[b]**	**Mean ± ΔS[c]**
Anthocyanins														
1	Delphinidin 3-*O*-glucoside-5-*O*-glucoside	18.69	22.10	23.42	22.83	23.57	22.12 *± 1.92	23.00	25.14	25.02	24.66	26.06	24.18 *± 1.18	23.45* ± 2.07
2	Cyanidin 3-*O*-glucoside-5-*O*-glucoside	4.11	5.50	5.79	5.59	5.82	5.36 *± 0.67	6.80	8.25	8.71	8.00	8.94	8.13 *± 0.80	6.75* ± 1.60
3	Delphinidin 3-*O*-glucoside	45.41	44.97	37.63	42.83	38.36	41.83 *± 3.46	44.56	42.89	33.48	41.42	33.57	39.18 *± 5.02	40.51* ± 4.42
4	Petunidin 3-*O*-glucoside-5-*O*-glucoside	10.05	12.12	13.08	12.56	12.41	12.04 *± 1.13	12.01	13.72	13.18	13.10	14.13	13.22 *± 0.77	12.63* ± 1.12
5	Peonidin 3-*O*-glucoside-5-*O*-glucoside	24.61	30.24	31.98	30.74	29.93	29.49 *± 2.74	30.31	33.73	35.53	33.63	35.66	33.77 *± 2.08	31.64* ± 3.23
6	Malvidin 3-*O*-glucoside-5-*O*-glucoside	103.07	120.75	131.68	127.65	123.23	121.27 *± 10.61	130.17	135.48	146.75	137.93	143.48	138.76 *± 6.91	130.02* ± 12.51
7	Cyanidin 3-*O*-glucoside	5.39	5.27	5.13	5.16	4.94	5.17 *± 0.18	4.89	4.88	4.33	4.80	4.47	4.67 *± 0.26	4.93* ± 0.34
8	Petunidin 3-*O*-glucoside	21.17	20.89	16.98	20.22	17.23	19.29 *± 1.93	19.63	18.27	14.98	19.14	16.63	17.72 *± 1.83	18.51* ± 2.01
9	Peonidin 3-*O*-glucoside	4.51	4.69	4.51	4.82	4.54	4.61 *± 0.16	4.22	4.59	4.27	5.01	4.14	4.44 *± 0.35	4.53* ± 0.29
10	Malvidin 3-*O*-glucoside	84.67	82.55	77.63	82.24	79.13	81.24 *± 3.62	85.43	79.69	73.50	88.00	78.14	80.95 *± 5.73	81.10* ± 4.67
11	Delphinidin 3-*O*-(6′′-*O*-acetyl)-glucoside	13.46	12.09	10.15	12.53	10.93	11.83 *± 1.25	13.34	11.83	9.99	13.87	11.05	12.01 *± 1.54	11.92* ± 1.37
12	Cyanidin 3-*O*-(6′′-*O*-acetyl)-glucoside	1.34	1.26	1.10	1.26	1.25	1.24 *± 0.09	1.48	1.30	1.12	1.48	1.26	1.32 *± 0.15	1.29* ± 0.13
13	Petunidin 3-*O*-(6′′-*O*-acetyl)-glucoside	2.38	2.18	1.95	2.19	2.05	2.14 *± 0.15	2.42	2.20	1.89	2.50	2.17	2.23 *± 0.23	2.19* ± 0.20
14	Petunidin 3-*O*-(6′′-*O*-acetyl)-glucoside-5-*O*-glucoside	1.86	1.89	1.97	1.91	1.86	1.89 *± 0.04	1.74	1.80	1.78	1.85	1.82	1.79 *± 0.04	1.85* ± 0.07
15	Delphinidin 3-*O*-(6′′-*O*-coumaryl)-glucoside	5.11	4.71	3.86	4.06	4.14	4.37 *± 0.50	4.65	3.85	3.17	4.16	3.63	3.89 *± 0.53	4.13* ± 0.57
16	Malvidin 3-*O*-(6′′-*O*-acetyl)-glucoside	8.53	8.48	8.20	8.70	8.51	8.48 *± 0.40	8.95	8.98	8.90	9.93	9.12	9.17 *± 0.44	8.83* ± 0.55
17	Malvidin 3-*O*-(6′′-*O*-coumaryl)-glucoside-5-*O*-glucoside	14.09	14.71	15.76	14.17	14.17	14.57 *± 0.92	15.77	15.11	15.79	14.28	15.46	15.28 *± 0.62	14.93* ± 0.85
18	Peonidin 3-*O*-(6′′-*O*-coumaryl)-glucoside-5-*O*-glucoside	2.87	3.25	3.56	3.25	3.10	3.20 *± 0.25	3.30	3.32	3.68	3.36	3.58	3.44 *± 0.17	3.33* ± 0.25
19	Peonidin 3-*O*-(6′′-*O*-acetyl)-glucoside	2.77	2.71	2.58	2.55	2.55	2.63 *± 0.10	2.93	2.75	2.68	3.12	2.74	2.84 *± 0.18	2.74* ± 0.18
20	Cyanidin 3-*O*-(6′′-*O*-coumaryl)-glucoside	2.30	2.06	1.75	1.88	1.91	1.97 *± 0.20	2.04	1.69	1.49	1.92	1.74	1.77 *± 0.20	1.88* ± 0.22
21	Petunidin 3-*O*-(6′′-*O*-coumaryl)-glucoside	2.11	2.03	1.63	1.75	1.71	1.84 *± 0.20	1.95	1.61	1.36	1.74	1.52	1.63 *± 0.21	1.74* ± 0.23
22	Delphinidin 3-*O*-(6′′-caffeoyl)-glucoside	1.31	1.25	1.16	1.19	1.24	1.23 *± 0.05	1.27	1.18	1.12	1.17	1.16	1.17 *± 0.05	1.21* ± 0.06
23	Peonidin 3-*O*-(6′′-*O*-coumaryl)-glucoside	2.22	2.11	1.91	1.97	2.07	2.05 *± 0.11	2.10	1.87	1.74	2.02	1.91	1.92 *± 0.13	1.99* ± 0.14
24	Malvidin 3-*O*-(6′′-*O*-coumaryl)-glucoside	8.88	9.32	8.18	8.53	8.59	8.69 *± 0.45	8.99	8.14	7.60	8.66	8.27	8.33 *± 0.53	8.51* ± 0.52
Subtotal		390.91	417.14	411.58	420.58	403.24		431.96	432.24	422.07	445.76	430.64		

Table S3 *Cont.*

		Wines												Mean ± ΔS[c]
		R1	R2	R3	R4	R5	Mean ± ΔS[a]	R1 LAB	R2 LAB	R3 LAB	R4 LAB	R5 LAB	Mean ± ΔS[b]	
Flavonols														
25	Myricetin-3-*O*-rutinoside	0.05	0.05	0.05	0.05	0.05	0.04 *± 0.00	0.06	0.04	0.05	0.05	0.04	0.04 *± 0.00	0.05* ± 0.00
26	Myricetin-3-*O*-glucoside	0.76	0.76	0.55	0.59	0.71	0.67 *± 0.09	0.79	0.71	0.43	0.58	0.68	0.63 *± 0.13	0.66 *± 0.11
27	Quercetin 3-*O*-glucuronide	0.03	0.03	0.03	0.03	0.02	0.02 *± 0.00	0.04	0.04	0.04	0.03	0.03	0.03 *± 0.00	0.03* ± 0.00
28	Isorhamnetin 3-*O*-glucoside	1.01	1.04	0.90	0.97	0.84	0.95 *± 0.08	1.07	0.97	0.80	0.89	0.91	0.92 *± 0.09	0.94* ± 0.09
29	Quercetin 3-*O*-glucoside	0.08	0.06	0.05	0.05	0.06	0.06 *± 0.01	0.06	0.07	0.05	0.05	0.07	0.06 *± 0.00	0.06* ± 0.01
30	Quercetin 3-*O*-rutinoside	0.10	0.09	0.07	0.08	0.08	0.08 *± 0.01	0.10	0.07	0.06	0.07	0.07	0.07 *± 0.01	0.08* ± 0.01
31	Dihydroquercetin 3-*O*-ramnoside	0.08	0.07	0.08	0.08	0.06	0.07 *± 0.00	0.07	0.07	0.07	0.06	0.06	0.06 *± 0.00	0.07* ± 0.01
Subtotal		2.10	2.11	1.74	1.85	1.81		2.18	1.96	1.50	1.74	1.86		
Flavan-3-ols														
32	Procyanidin B1	21.63	18.14	16.65	19.71	17.47	18.72 *± 1.89	33.82	34.79	24.90	28.64	27.69	29.96 *± 3.99	24.34* ± 6.52
33	Procyanidin B-type 1	5.57	4.02	3.07	4.26	3.19	4.02 ± 0.95	6.21	4.81	3.07	4.40	3.59	4.41 ± 1.15	4.22 ± 1.05
34	Procyanidin C1	5.00	4.32	3.85	4.10	4.15	4.28 *± 0.41	5.59	5.09	3.75	4.35	3.75	4.50 *± 0.78	4.40* ± 0.62
35	(+) catechin	36.43	30.40	29.97	32.22	30.98	32.00 *± 2.56	48.76	51.70	39.79	44.44	40.76	45.09 *± 4.99	38.55* ± 7.75
36	Procyanidin C-type 1	0.66	0.45	0.43	0.54	0.56	0.52 ± 0.09	2.14	2.18	1.64	1.70	1.47	1.82 *± 0.30	1.18* ± 0.70
37	Procyanidin B-type 2	1.61	1.18	0.94	1.36	1.20	1.25 *± 0.23	2.05	1.85	1.29	1.76	1.41	1.67 ± 0.29	1.46* ± 0.34
38	Procyanidin B2	18.58	13.79	13.25	15.41	12.38	14.68 ± 2.32	20.40	18.81	13.22	16.82	12.61	16.37 ± 3.30	15.53 ± 2.91
39	(-) epicatechin	18.82	13.78	14.39	15.29	13.56	15.17 *± 2.07	21.28	19.75	15.25	17.21	14.94	17.68 *± 2.64	16.43* ± 2.65
40	Procyanidin C-type 2	1.88	1.38	1.64	1.50	1.38	1.55 *± 0.20	2.03	2.21	1.22	1.98	1.83	1.85 *± 0.35	1.71* ± 0.32
41	Procyanidin C-type 3	3.64	2.55	2.35	2.68	2.53	2.75 ± 0.49	4.25	3.93	2.58	3.40	2.85	3.40 ± 0.66	3.08 ± 0.66
42	Procyanidin B-type 3	22.04	23.52	25.76	25.56	24.35	24.24 *± 1.62	23.45	29.27	24.85	25.27	23.71	25.30 *± 2.81	24.78* ± 2.30
43	Procyanidin B-type 4	3.15	2.35	2.19	2.73	2.23	2.53 *± 0.39	3.14	2.84	2.00	2.33	2.22	2.50 *± 0.44	2.52* ± 0.41
Subtotal		139.02	115.88	114.49	125.37	113.98		173.13	177.23	133.55	152.30	136.81		
Phenolic acids														
44	Gallic acid	20.51	17.28	17.13	18.58	19.55	18.61 *± 1.53	23.59	20.84	19.49	21.79	20.54	21.25 *± 1.56	19.93* ± 2.03
45	Protocatechuic acid	1.09	1.27	1.23	1.37	1.24	1.23 *± 0.10	1.63	1.74	1.53	1.78	1.66	1.66 *± 0.10	1.45* ± 0.24
46	Caftaric acid	15.04	15.71	16.35	15.00	15.56	15.53 *± 0.87	3.71	3.22	1.92	2.78	4.05	3.13 *± 0.78	9.33 ± 6.41
47	Coutaric acid	8.03	8.66	9.07	8.23	8.68	8.53 *± 0.41	1.52	1.19	0.73	1.18	1.61	1.24 *± 0.32	4.89 ± 3.76
48	Caffeic acid	0.22	0.21	0.25	0.27	0.27	0.24 *± 0.02	4.33	4.26	4.38	4.24	4.37	4.31 *± 0.10	2.28 ± 2.09

Table S3 *Cont.*

		Wines												
		R1	R2	R3	R4	R5	Mean ± ΔS[a]	R1 LAB	R2 LAB	R3 LAB	R4 LAB	R5 LAB	Mean ± ΔS[b]	Mean ± ΔS[c]
49	Ferulic acid	0.08	0.08	0.06	0.06	0.05	0.06 ± 0.00	0.03	0.03	0.00	0.01	0.01	0.01 *± 0.01	0.04 ± 0.03
50	p-Coumaric acid	0.20	0.28	0.68	0.74	0.57	0.49 ± 0.22	2.65	2.90	3.37	2.99	2.96	2.97 *± 0.25	1.73 ± 1.29
51	Coumaric acid	0.03	0.04	0.04	0.05	0.05	0.04 *± 0.00	0.62	0.68	0.74	0.72	0.71	0.69 *± 0.04	0.37 ± 0.34
Subtotal		45.20	43.52	44.81	44.29	45.98		38.09	34.86	32.16	35.49	35.90		
Stilbenes														
52	Trans-piceid	1.27	1.12	1.19	1.21	0.90	1.13 *± 0.13	0.60	0.38	0.30	0.35	0.28	0.38 *± 0.12	0.76* ± 0.41
53	Cis-piceid	6.00	5.81	5.85	5.80	4.71	5.63 *± 0.50	5.48	4.55	4.21	4.16	3.91	4.46 *± 0.58	5.05* ± 0.8
54	Trans-resveratrol	0.32	0.31	0.32	0.30	0.28	0.30 *± 0.01	0.52	0.49	0.45	0.43	0.43	0.46 *± 0.03	0.39* ± 0.09
55	Cis-resveratrol	2.72	2.79	2.82	2.83	2.34	2.69 *± 0.20	3.15	3.13	3.38	2.66	2.81	3.02 *± 0.27	2.86* ± 0.29
Subtotal		10.31	10.04	10.17	10.13	8.24		9.75	8.55	8.35	7.60	7.43		
Total		587.54	588.68	582.79	602.22	573.25		655.12	654.84	597.64	642.90	612.64		

R1-R5-wines from the Rondo variety, in which alcoholic fermentation was carried out by different yeast strains and malolactic fermentation was spontaneous; R1 LAB-R5 LAB-wines from the Rondo variety, in which alcoholic fermentation was carried out by different yeast strains (but the same as in R1-R5 wines), and malolactic fermentation was induced

a - (*) in the same row of Table S2 and Table S3 indicates significant difference between the means of Z!-Z5 and R1-R5 wines ($p < 0.0001$)

b - (*) in the same row of Table S2 and Table S3 indicates significant difference between the means of Z1 LAB-Z5 LAB and R1 LAB-R5 LAB wines ($p < 0.0001$)

c – (*) in the same row of Table S2 and Table S3 indicates mean significantly different among the wines from two selected grape varieties with $p < 0.000001$ (only for procyaninin C-type 1. $p = 0.0337$)

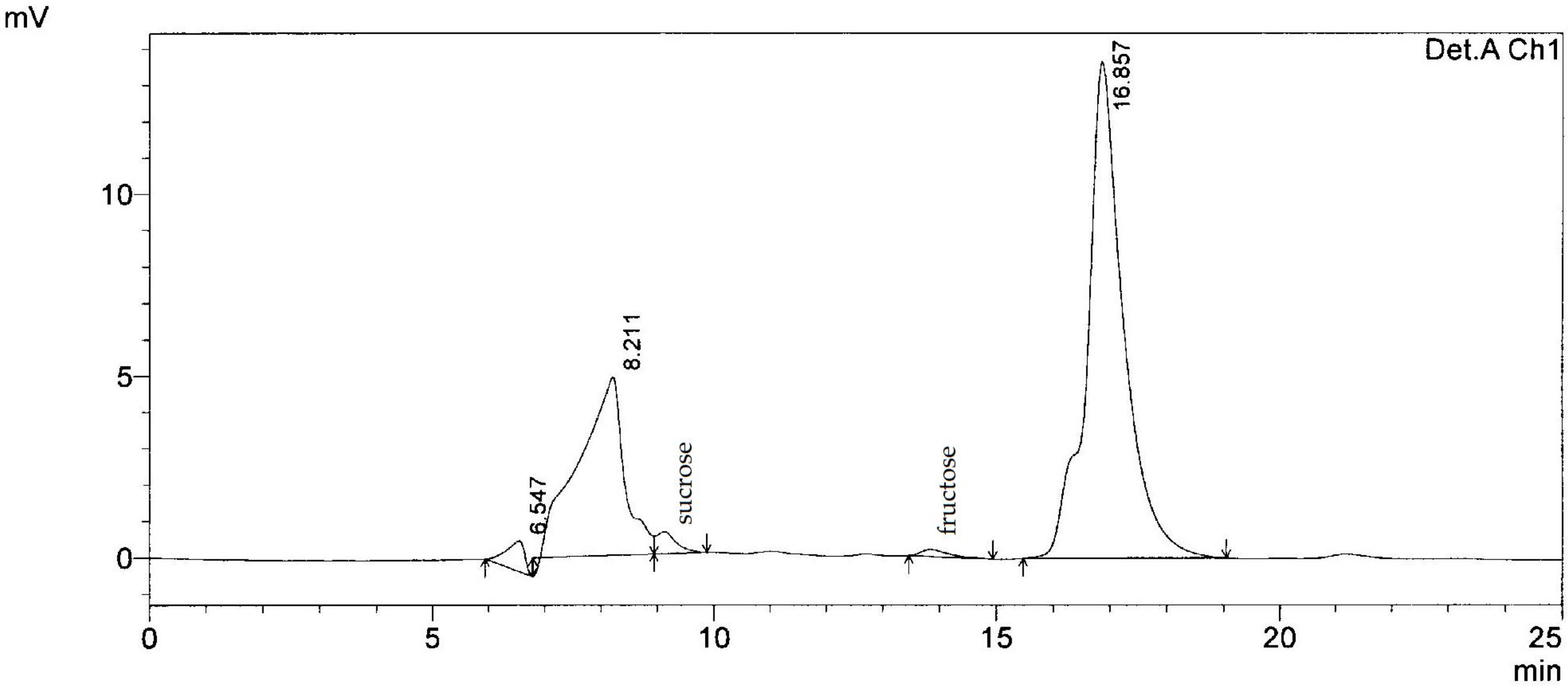

Figure S1. Chromatogram of sugars in Rondo wine

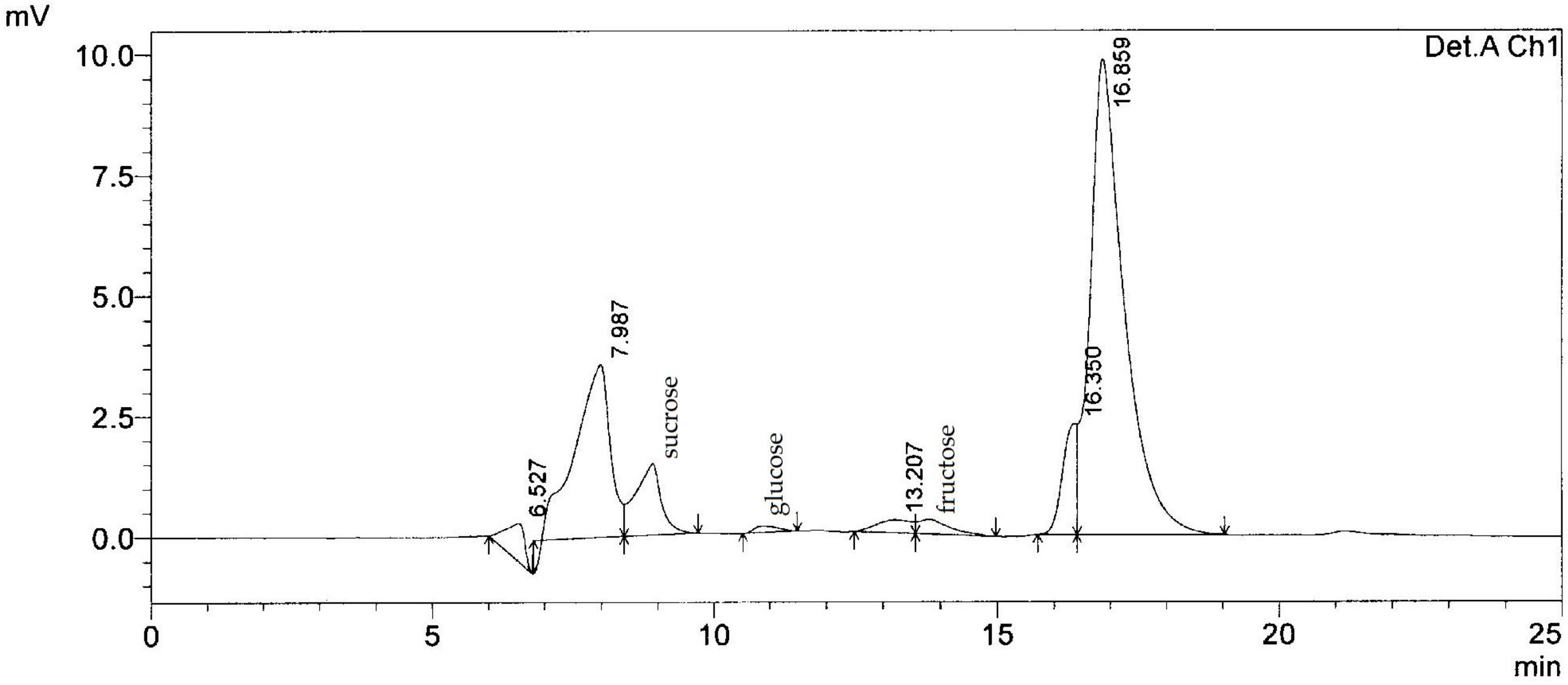

Figure S2. Chromatogram of sugars in Zweigelt wine

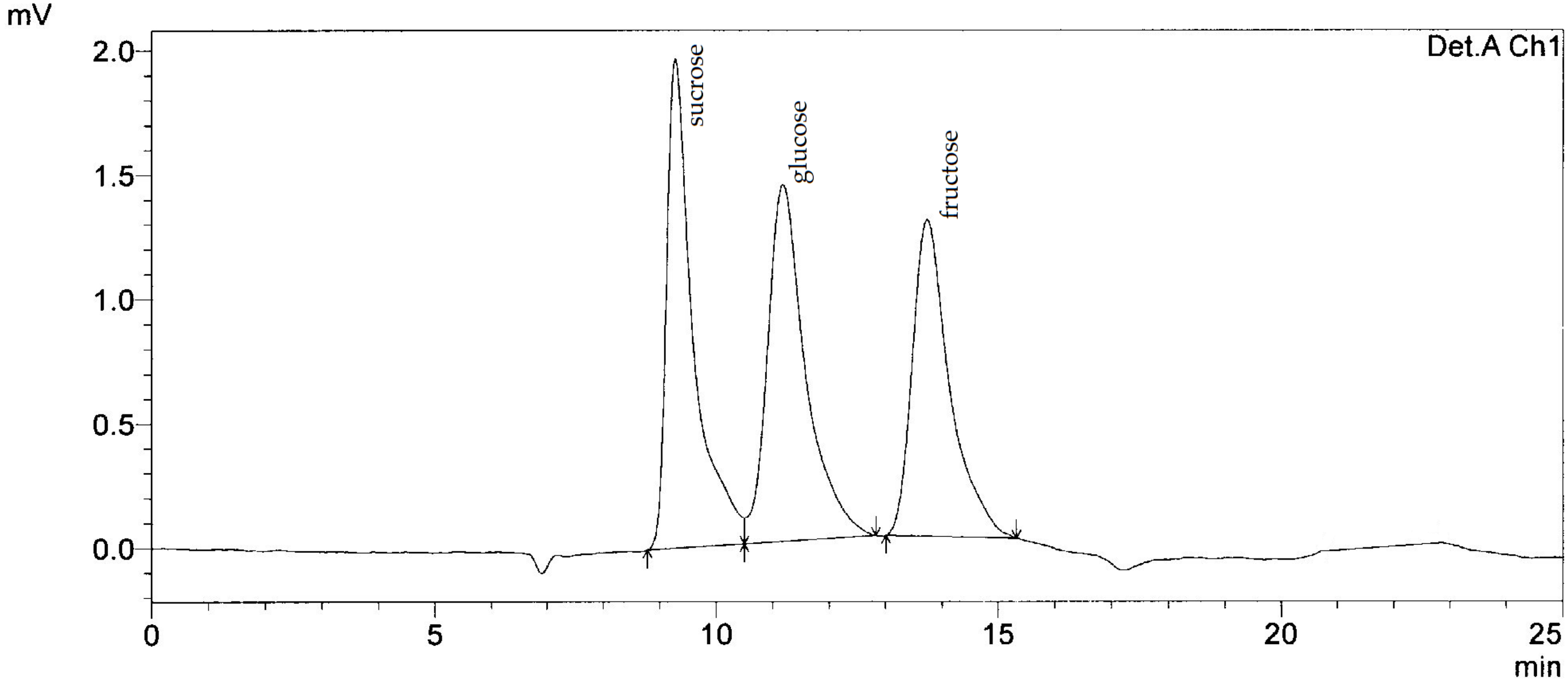

Figure S3. Chromatogram of sugars standards

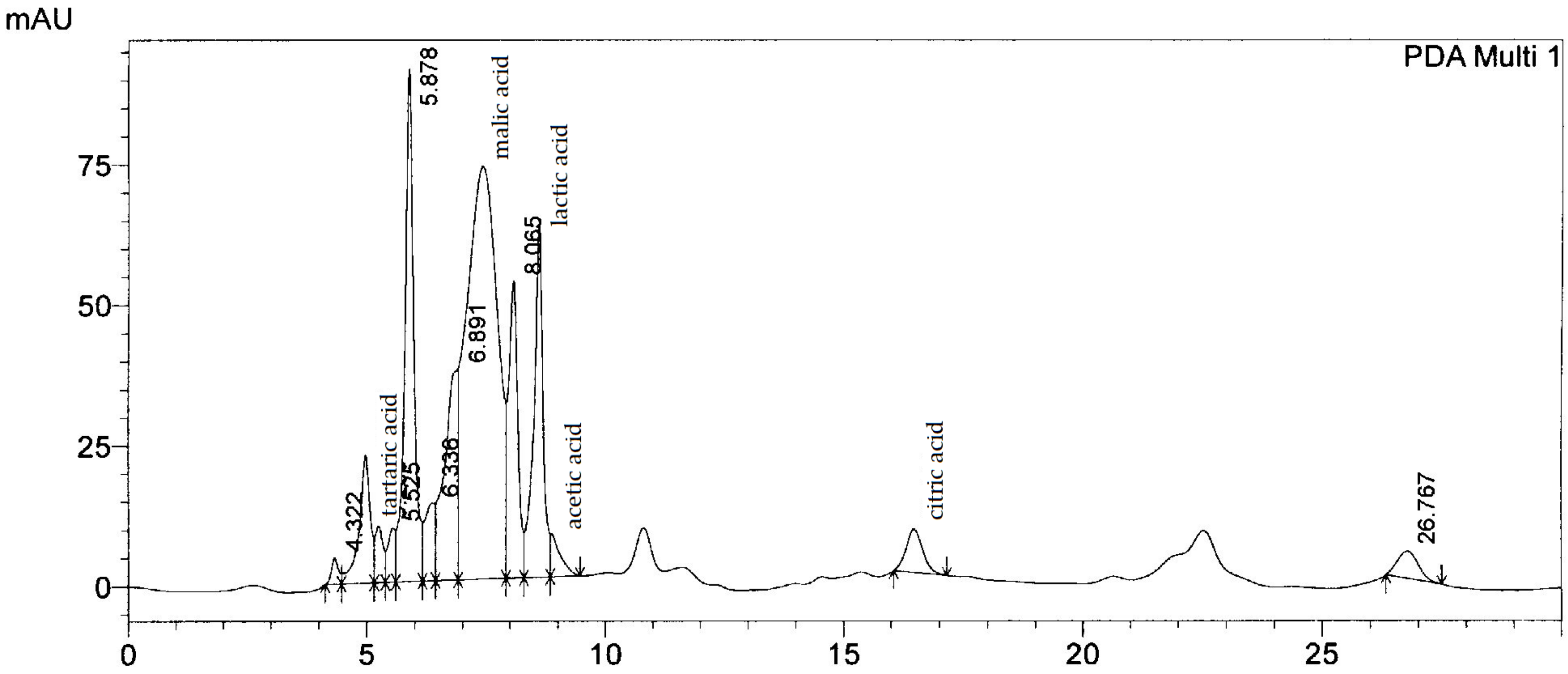

Figure S4. Chromatogram of organic acids in Rondo wine

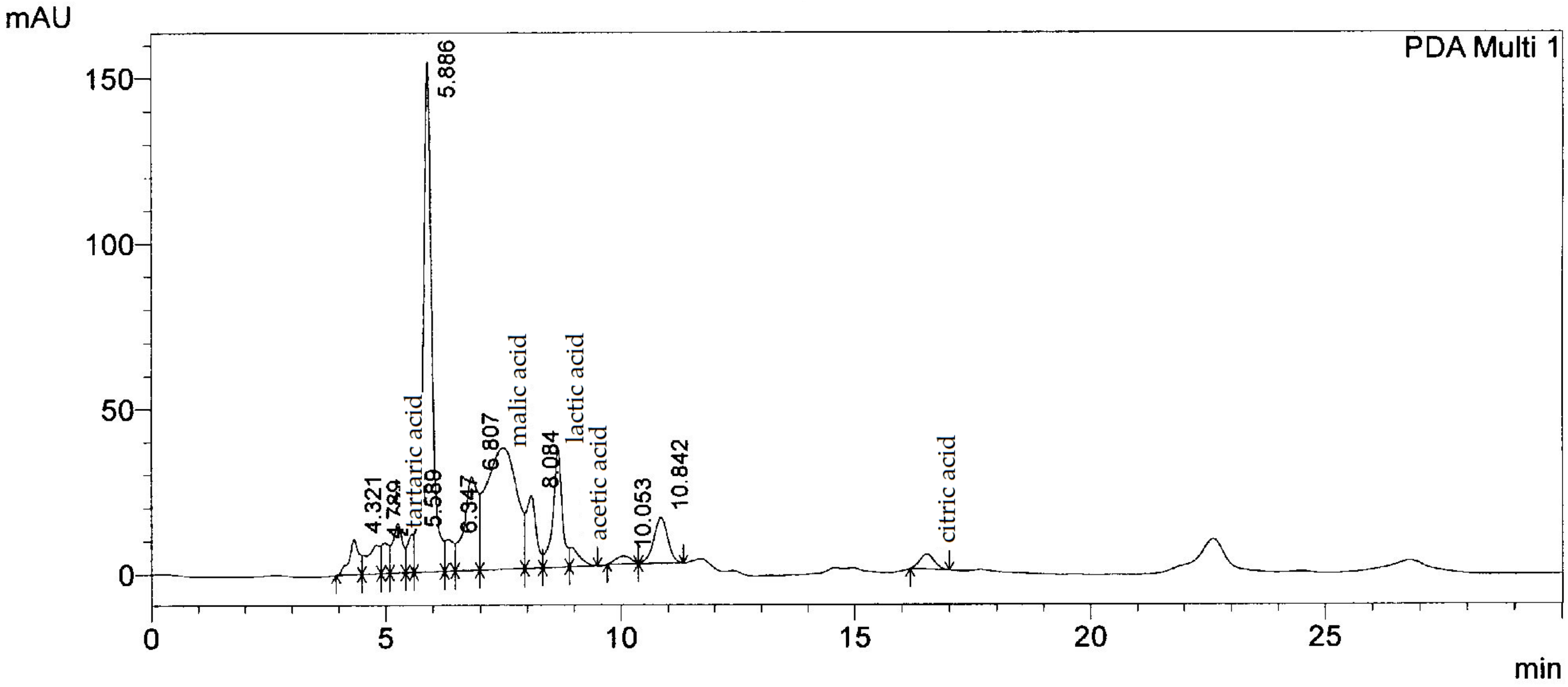

Figure S5. Chromatogram of organic acids in Zweigelt wine

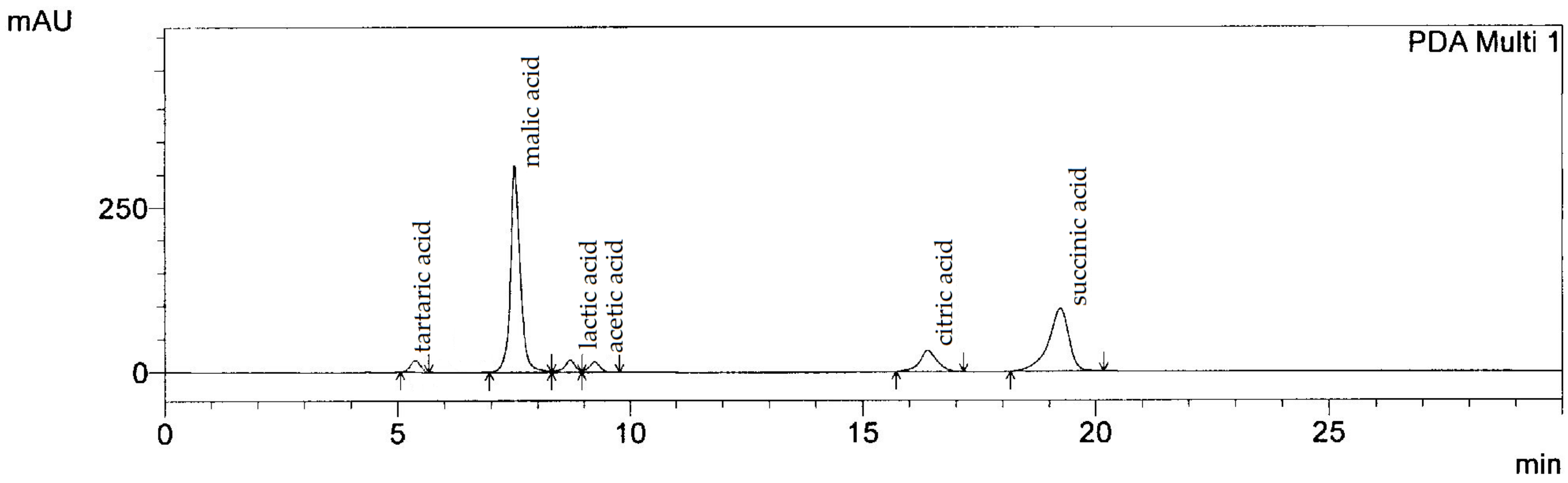

Figure S6. Chromatogram of organic acid standards

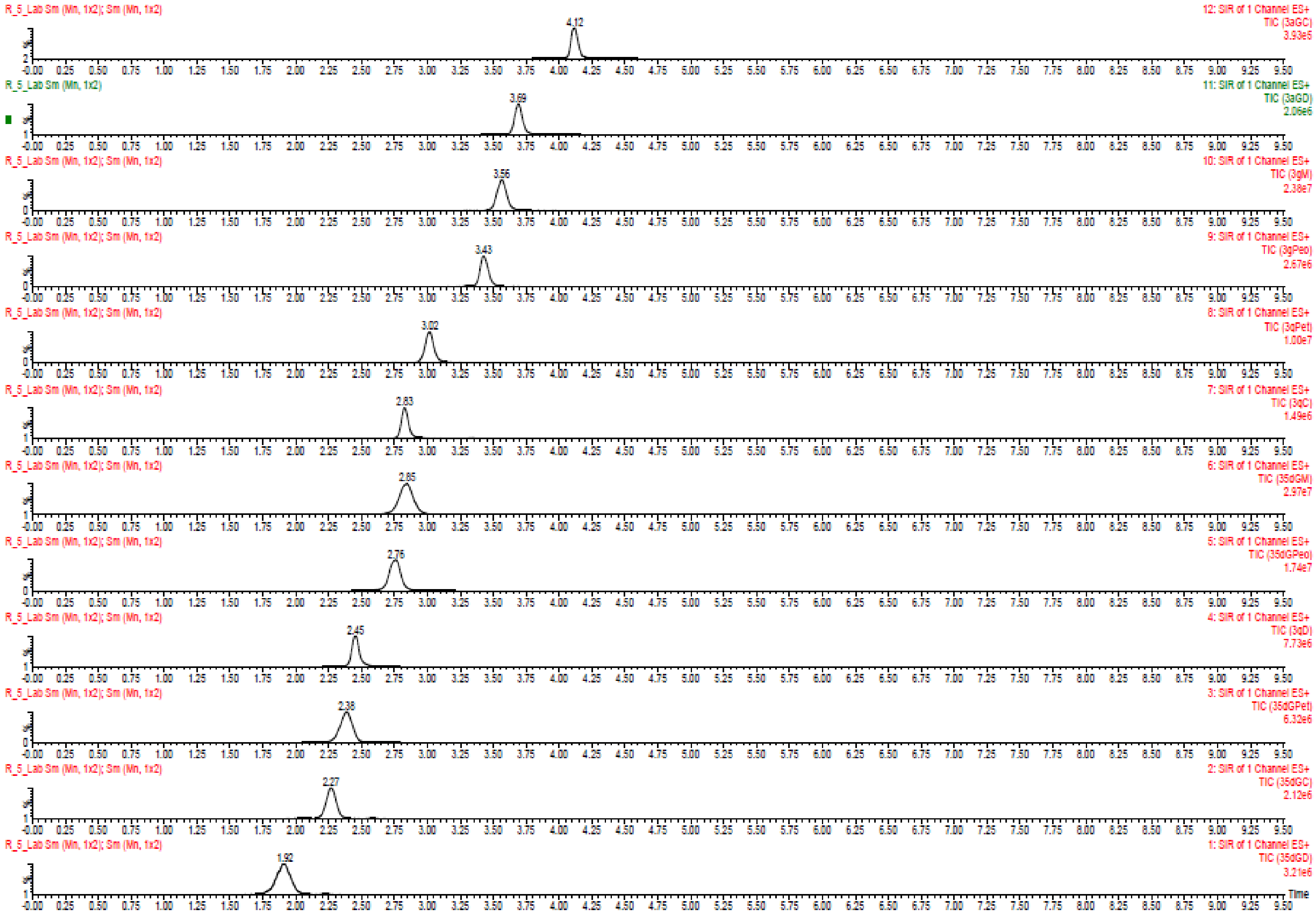

Figure S7. Single ion recording (SIR) of anthocyanins in Rondo wine detected by UPLC-MS/MS (part 1)

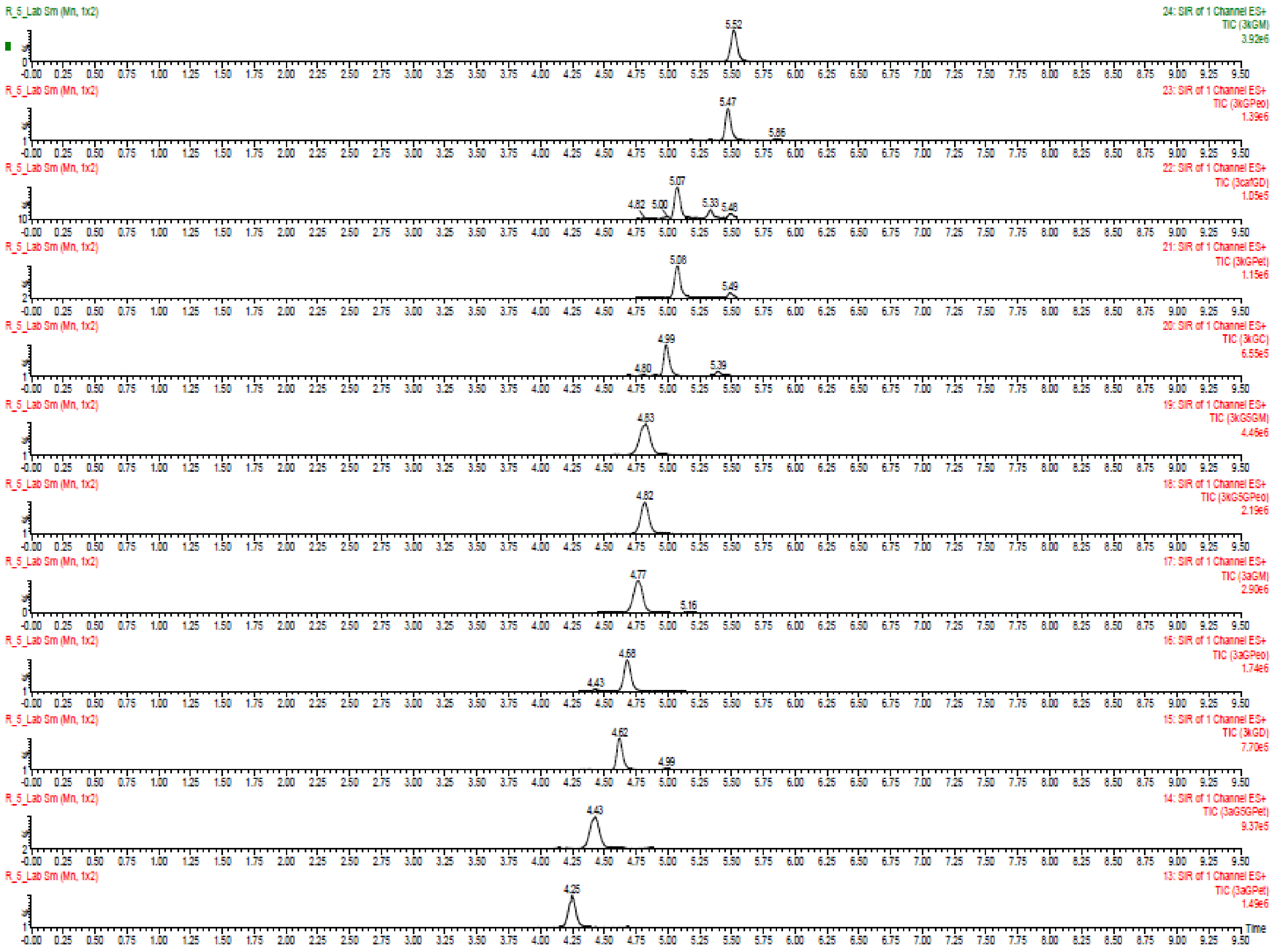

Figure S8. Single ion recording (SIR) of anthocyanins in Rondo wine detected by UPLC-MS/MS (part 2)

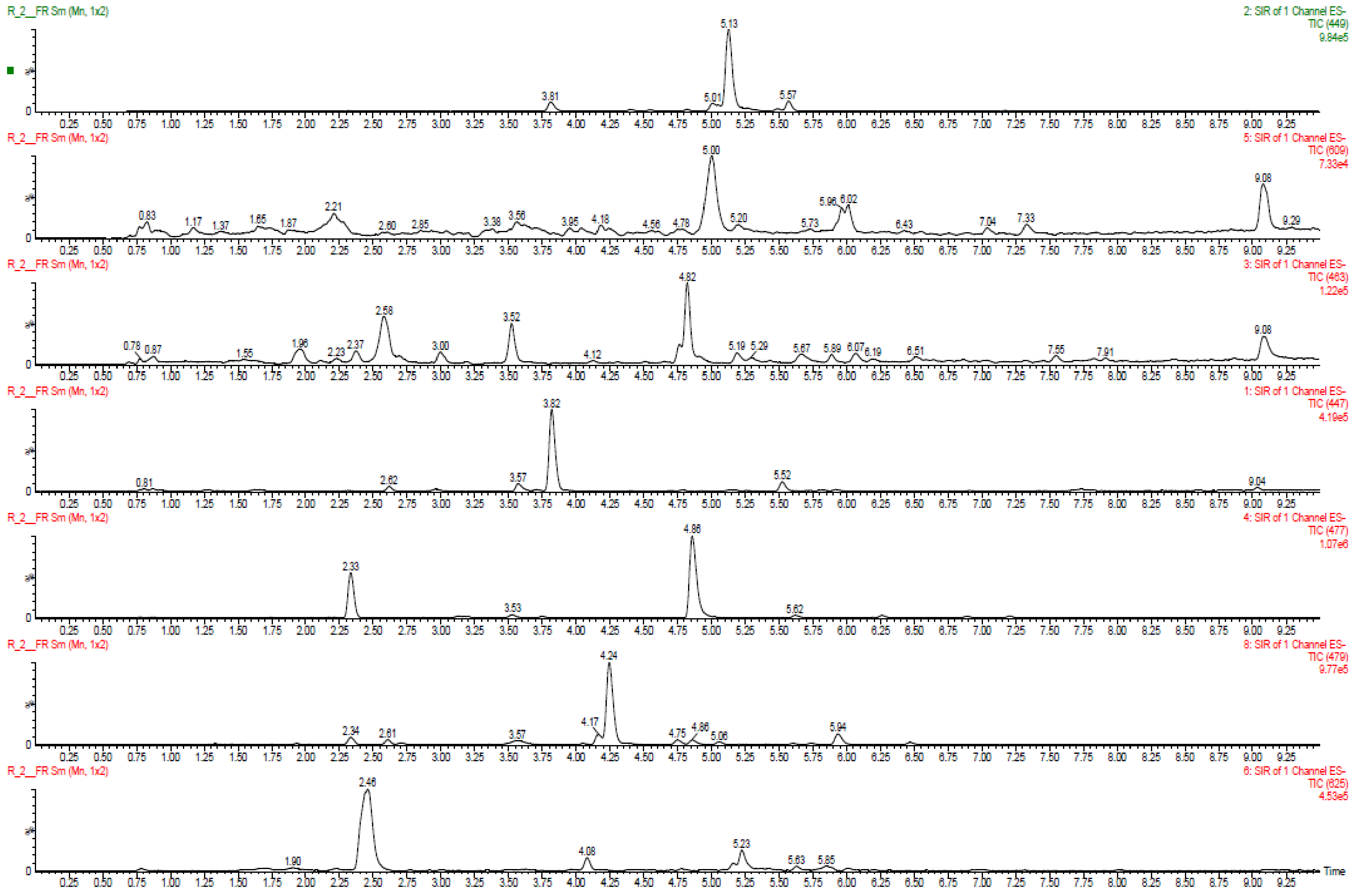

Figure S9. Single ion recording (SIR) of flavonols in Rondo wine detected by UPLC-MS/MS

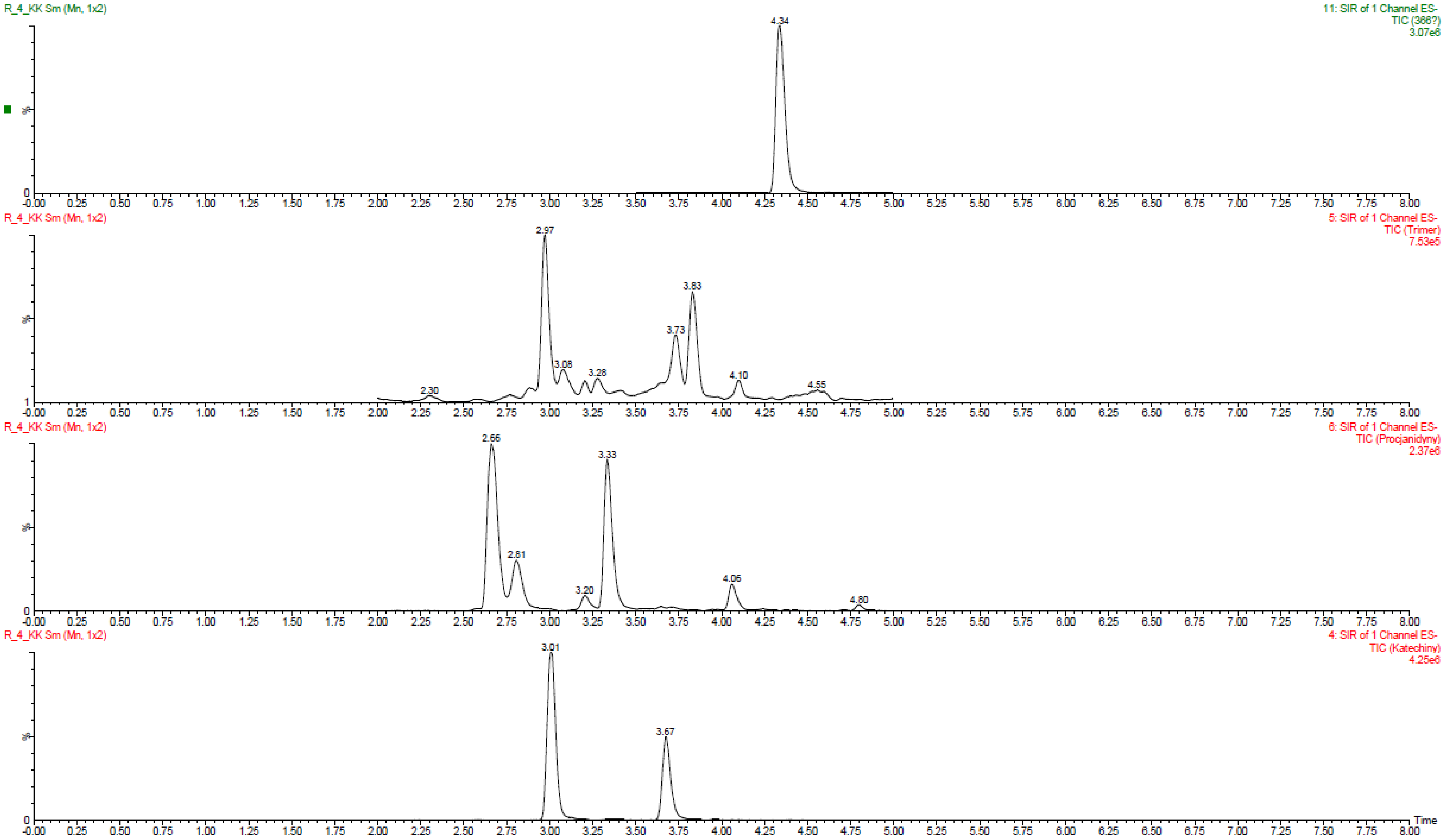

Figure S10. Single ion recording (SIR) of flavan-3-ols in Rondo wine detected by UPLC-MS/MS

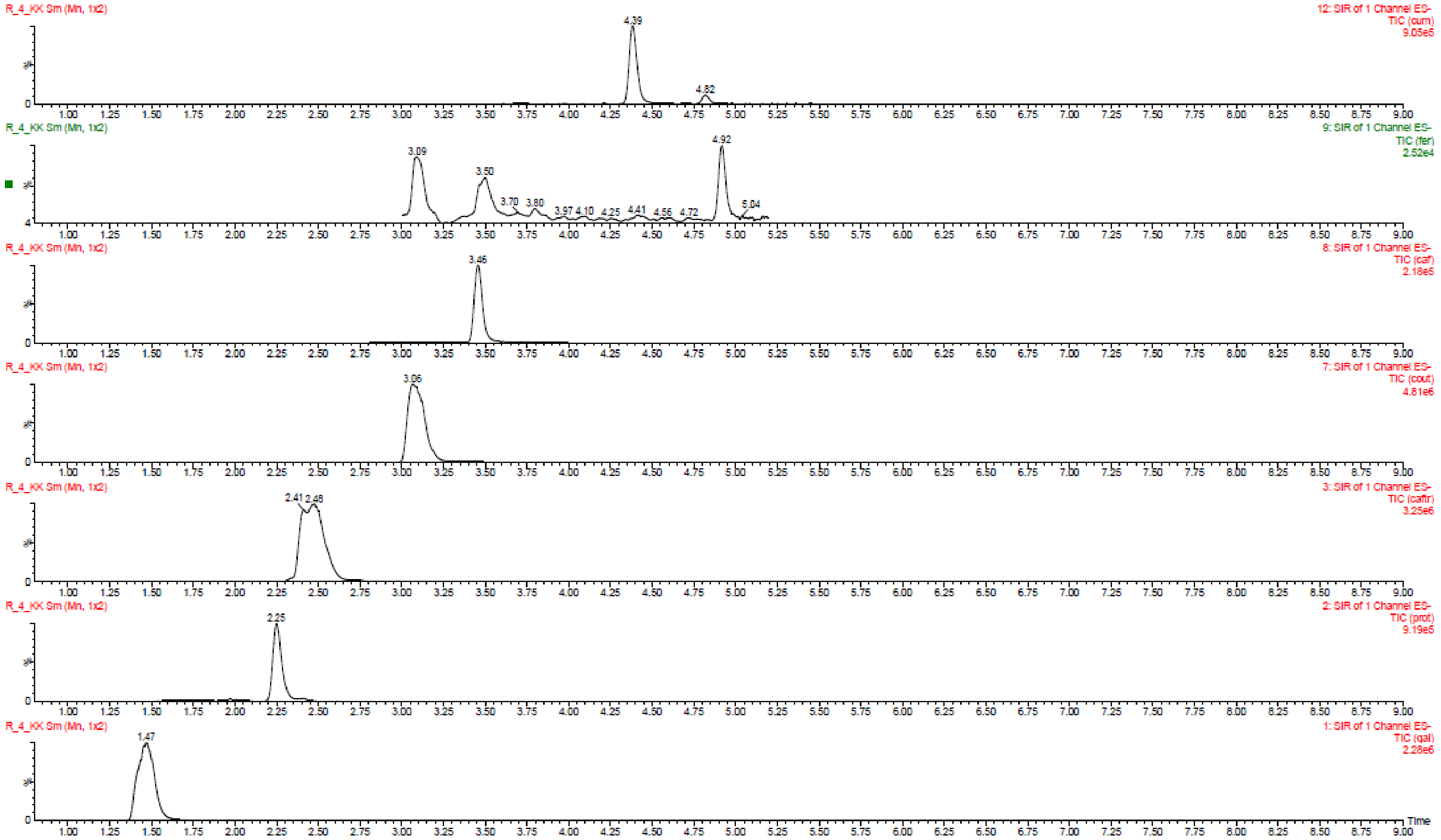

Figure S11. Single ion recording (SIR) phenolic acids in Rondo wine detected by UPLC-MS/MS

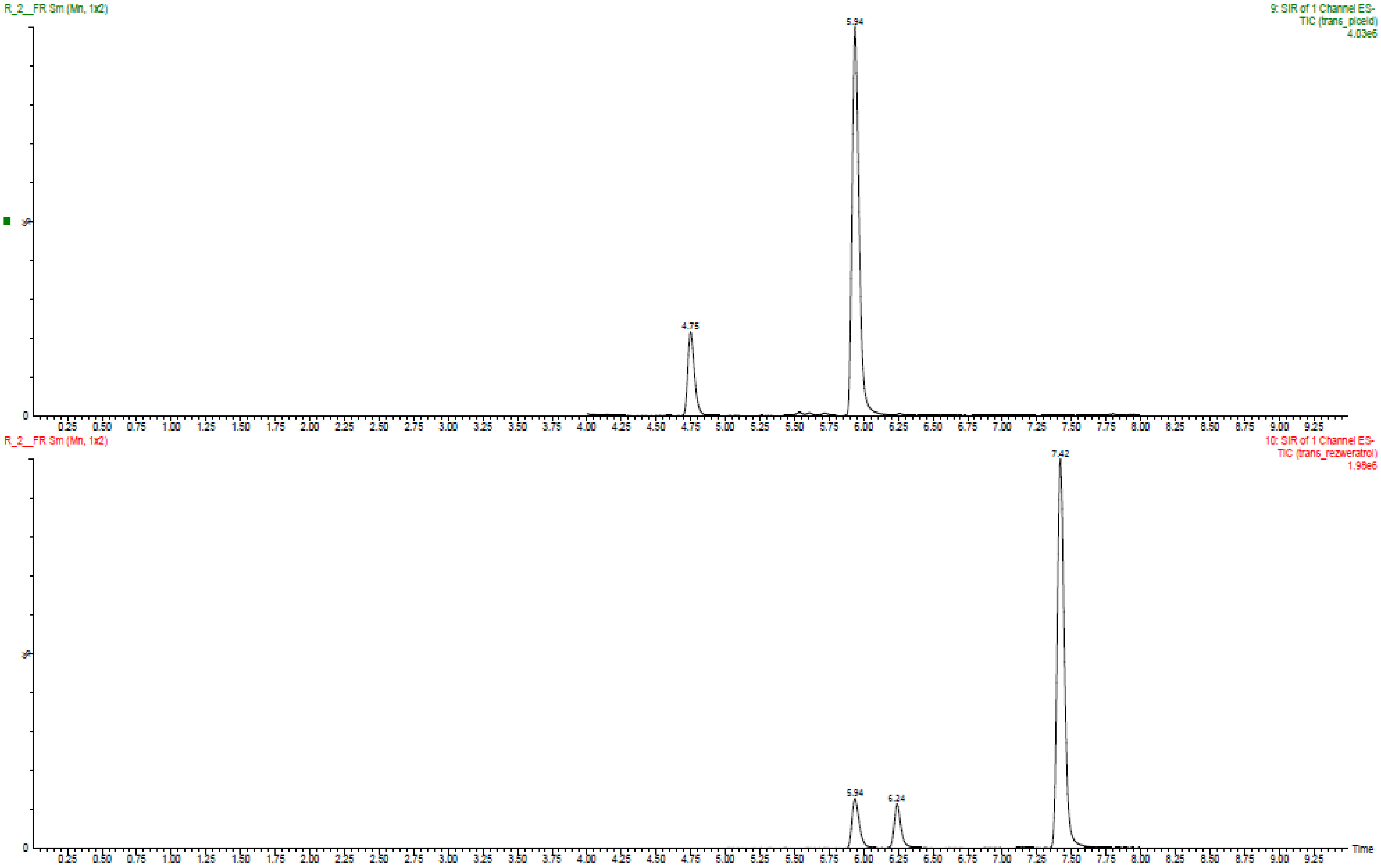

Figure S12. Single ion recording (SIR) of stilbenes in Rondo wine detected by UPLC-MS/MS
